# An Intrinsically Magnetic Epicardial Patch for Rapid Vascular Reconstruction and Drug Delivery

**DOI:** 10.1002/advs.202303033

**Published:** 2023-11-14

**Authors:** Bei Qian, Ao Shen, Shixing Huang, Hongpeng Shi, Qiang Long, Yiming Zhong, Zhaoxi Qi, Xiaojun He, Yecen Zhang, Wangxi Hai, Xinming Wang, Yanna Cui, Ziheng Chen, Huixia Xuan, Qiang Zhao, Zhengwei You, Xiaofeng Ye

**Affiliations:** ^1^ Department of Cardiovascular Surgery, Ruijin Hospital Shanghai Jiaotong University School of Medicine Shanghai 200025 China; ^2^ State Key Laboratory for Modification of Chemical Fibers and Polymer Materials College of Materials Science and Engineering Institute of Functional Materials Research Base of Textile Materials for Flexible Electronics and Biomedical Applications (China Textile Engineering Society) Shanghai Engineering Research Center of Nano‐Biomaterials and Regenerative Medicine Donghua University Shanghai 201620 China; ^3^ Department of Nuclear Medicine, Ruijin Hospital Shanghai Jiaotong University School of Medicine Shanghai 200025 China; ^4^ Department of Pharmacology and Chemical Biology Shanghai Jiaotong University School of Medicine Shanghai 200000 China; ^5^ School of Mechatronics Engineering and Automation Shanghai University Shanghai 200000 China

**Keywords:** epicardial patch, magnetic field therapy, myocardial infarction, myocardial revascularization, targeted drug delivery

## Abstract

Myocardial infarction (MI) is a major cause of mortality worldwide. The major limitation of regenerative therapy for MI is poor cardiac retention of therapeutics, which results from an inefficient vascular network and poor targeting ability. In this study, a two‐layer intrinsically magnetic epicardial patch (MagPatch) prepared by 3D printing with biocompatible materials like poly (glycerol sebacate) (PGS) is designed, poly (ε‐caprolactone) (PCL), and NdFeB. The two‐layer structure ensured that the MagPatch multifariously utilized the magnetic force for rapid vascular reconstruction and targeted drug delivery. MagPatch accumulates superparamagnetic iron oxide (SPION)‐labelled endothelial cells, instantly forming a ready‐implanted organization, and rapidly reconstructs a vascular network anastomosed with the host. In addition, the prefabricated vascular network within the MagPatch allowed for the efficient accumulation of SPION‐labelled therapeutics, amplifying the therapeutic effects of cardiac repair. This study defined an extendable therapeutic platform for vascularization‐based targeted drug delivery that is expected to assist in the progress of regenerative therapies in clinical applications.

## Graphical abstract

The intrinsically magnetic epicardial patch (MagPatch) is designed for rapid vascular reconstruction and targeted drug delivery in myocardial infarction therapy. MagPatch utilizes superparamagnetic iron oxide (SPION)‐labelled endothelial cells to form a functional vascular network and accumulates SPION‐labelled drugs for enhancing therapeutic effects. This technology offers a promising platform for vascularization‐based targeted drug delivery in regenerative medicine.

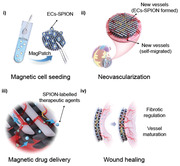

## 1 Introduction

Myocardial infarction (MI) is the predominant cause of global cardiovascular mortality.^[^ ^1^ ^]^ Insufficient blood perfusion to the ischemic zone causes myocardial necrosis and fibrotic scar formation, resulting in ventricular dysfunction and heart failure (HF).^[^ ^2^ ^]^ In recent years, with increasing insight into the pathophysiological processes of MI, novel therapeutic agents have been investigated to enhance the repair of damaged myocardium, including nanovesicles (NVs),^[^ ^3^ ^]^ adenovirus (Ad)^[^ ^4^ ^]^ and mRNA,^[^ ^5^ ^]^ holding promise for preventing HF and improving the prognosis after MI. NVs are tiny particles that can be loaded with therapeutic molecules such as drugs or genetic material. NVs are generated by cells that play a crucial role in intercellular communication.^[^ ^6^ ^]^ These nanoscale vesicles can carry a variety of therapeutic cargo, including proteins, nucleic acids, and drugs.^[^ ^7^ ^]^ In the context of MI, exosomes are being investigated for their ability to deliver regenerative factors and genetic material to the damaged heart tissue, promoting tissue repair and reducing adverse remodeling.^[^ ^8^ ^]^ Ad is one of gene therapy tool and involves the use of modified adenoviruses as vehicles for delivering therapeutic genes to the heart cells.^[^ ^4^ ^]^ These engineered viruses can efficiently enter the cardiac cells and introduce therapeutic genes that promote tissue repair and regeneration. Ad therapy is promising for enhancing myocardial healing and preventing adverse remodeling following MI.^[^ ^9^ ^]^ Like Ad, mRNA is another emerging gene therapy tool in the treatment of MI.^[^ ^10^ ^]^ By utilizing mRNA molecules, such as VEGF,^[^ ^5^ ^]^ researchers can provide specific instructions to heart cells and stimulate them to produce therapeutic proteins or factors that aid in tissue repair and regeneration. This technology can potentially enhance cardiac function and limit adverse remodeling post‐MI.

Although NVs, Ad, and mRNA therapies show promise for the treatment of myocardial infarction, their clinical application is hindered by the need to improve the targeted delivery and retention of these therapeutic agents within the damaged heart tissue.^[^ ^11^ ^]^ Conventional therapeutic approaches for drug delivery are limited by several hurdles. On the one hand, massive occlusion of blood circulation pathways in the infarcted area leads to blockage of the major route for drug accumulation, resulting in a low residence rate via intravenous injection of these agents.^[^ ^12^ ^]^ Although intramyocardial injection enables direct delivery of the drug to the infarcted myocardium, it is an invasive method with certain risks and is unsuitable for repeated injections over a short period.^[^ ^13^ ^]^ However, these novel therapeutic agents exhibit poor targeting capabilities. Previous studies have attempted several promising ligands to direct drugs to the damaged myocardium.^[^ ^14^ ^]^ Unfortunately, to date, no effective targeting ligands with wide acknowledgment can perfectly ensure a specific interaction of drugs at the targeted ischemic site.^[^ ^15^ ^]^

These obstacles lead to extremely low drug concentrations in the infarcted myocardium, resulting in an insufficient therapeutic dose, preventing these agents from playing a regulatory role after MI, and hindering their further clinical application.^[^ ^16^ ^]^ Therefore, an integrated drug delivery system is urgently required to build a vascular route in the infarcted area and subsequently allow abundant multidose delivery of therapeutic agents.

Poly (glycerol sebacate) (PGS) and poly (ε‐caprolactone) (PCL) are widely used as FDA‐approved biocompatible materials.^[^ ^17^ ^]^ In a previous study, hybrid PGS‐PCL scaffolds were designed and prepared using 3D printing to predict heart function after MI.^[^ ^18^ ^]^ NdFeB is a permanent magnet that is a fundamental component of biomedical systems.^[^ ^19^ ^]^ Wireless cells and drug delivery systems driven by magnetic fields usually focus on the therapeutic effect of external magnetic fields on target organs, especially superficial organs such as the skin and muscles.^[^ ^20^ ^]^ However, when applied to deep organs in the chest, such as regenerative therapy to the myocardium post‐MI, the off‐target effects of magnetic field‐mediated drug transport cannot be ignored.

In this study, we designed an intrinsically magnetic epicardial patch (MagPatch) with multifarious magnetic force utilization for rapid vascularization and replenishable‐targeted delivery. To ensure that the cells and drugs were accurately delivered to the heart area, the MagPatch was designed as a double‐layered structure. The PGS‐NdFeB layer was composed of PGS and NdFeB, providing a magnetic field. The PGS‐PCL layer comprised PGS and PCL, shielding the magnetic field. MagPatch can create a local magnetic field to efficiently accumulate endothelial cells (ECs) labeled with SPION in vitro. When implanted into the epicardium, MagPatch can build a vascular network connected with the host and serve as a platform for the magnetic capture of SPION‐labelled therapeutic agents, including NVs‐SPION and Ad‐SPION. Thus, we developed an extendable therapeutic platform for revascularization and magnetic accumulation of therapeutic agents for the treatment of MI, which is expected to be adapted to other ischemic diseases in the future.

## 2 Results

### 2.1 Morphology of MagPatch

**Figure 1:**
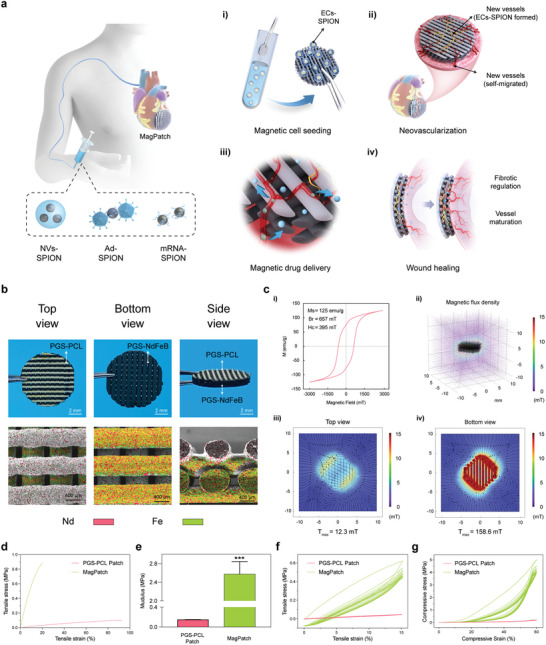
Principle application and characteristic of the MagPatch. a) Schematic of the MagPatch therapy. i) Magnetic cell transplantation: the ECs‐SPION accumulated actively on MagPatch via magnetic force; ii) Neovascularization: the integration of ECs‐SPION formed vascular‐like structure and self‐migrated vessels; iii) Magnetic drug delivery: MagPatch captured the SPION labelled drugs; iv) Wound healing: the accumulated drugs exerted therapeutic effects including fibrotic regulation and vessel maturation. b) Representative photographs and elemental mapping images of MagPatch. c) Computer simulation of the magnetic field generated by MagPatch. i) VSM curve of MagPatch; ii) Spatial magnetic field distribution of MagPatch; iii, iv) Planar magnetic field distribution of top and bottom view. d) Typical stress–strain curves of ultimate tensile tests. e) Young's modulus of PGS‐PCL patch and MagPatch in tensile tests (*n* = 3). f) Cyclic tensile tests at a strain of 15% for ten cycles. g) Compression tests at a strain of 60% for ten cycles. Data in (e) are presented as mean ± standard deviation and analyzed using one‐way ANOVA followed by Bonferroni's post hoc test. Statistical significance was indicated as follows: ***p < 0.001 compared with the PCL‐PGS Patch group. SPION, Superparamagnetic iron nanoparticle; ECs, endothelial cells; NVs, nanovesicles; Ad, adenovirus.

Each MagPatch was evenly 3D‐printed (Figures S1 and S2a, Supporting Information) and flexible with a multilayered framework (Movie S1, Supporting Information). The three bottom layers were black PGS‐NdFeB, and the top was white PGS‐PCL (**Figure** **1**a and Movie S2, Supporting Information) Elemental mapping revealed a uniform spatial distribution of Nd and Fe concentrated in black lays (Figure 1b; Figure S2c, Supporting Information). As seen in the scanning electron microscope (SEM) images, the center‐to‐center distance between filaments was ≈700 µm, and the diameter of the filaments of the PGS‐PCL patch was ≈440 µm. The diameters of about the MagPatch filaments were ≈420 and 470 µm, respectively. The SEM images showed that the patches had stacked constructions, with regular crisscrossed filaments. In the PGS‐PCL layer of the MagPatch, interconnected micropores were evenly distributed the whole filaments (Figure 1b), while the PGS‐NdFeB layer had smooth filaments and abundant interconnected micropores.

### 2.2 Magnetic Properties

The hysteresis loop of the MagPatch was measured by a vibrating sample magnetometer (VSM) (Figure 1c). The MagPatch had high values of saturation magnetization (M_s_) (125 emu g^−1^), magnetic remanence (B_r_) (657 mT) and coercivity (H_c_) (395 mT). The maximum energy product (BH)_max_ (22.5 kJ m^−3^) was obtained through calculation.^[^ ^21^ ^]^ The magnetic flux density was higher at the bottom (Figure 1c), which was consistent with the MagPatch structure. In the top view, the maximum magnetic flux density was only 12.3 mT (Figure 1c), while that in the bottom view was 158.6 mT (Figure 1c). Different magnetic field distribution caused SPION‐labelled cells and therapeutic agents to cluster in specific locations. In addition, as measured by a hand‐held Gaussian meter, the maximum magnetic field intensity of the top surface was 2.301 mT, while that of the bottom surface was 58.141 mT. This indicated that the MagPatch showed stable magnetic performance and that the bottom surface of the MagPatch could meet the requirements to serve as a platform for the magnetic capture of SPION‐labelled cells and therapeutic agents.^[^ ^22^ ^]^ Furthermore, in order to access the longevity of magnetic force in vivo, we conducted measurements on the intensity of magnetic field generated by MagPatch 42 days post‐implantation. The results indicated the intensity of magnetic field remains similar to those prior to implantation (55.09 ± 2.61 mT prior to implantation versus 53.42 ± 4.05 mT 28 days post in vivo implantation).

### 2.3 Mechanical Property

The mechanical properties of PGS‐PCL and MagPatch patches were measured using tensile and compression tests. The uniaxial tensile tests (Figure 1d,e) revealed a Young’ s modulue (*E*) of 0.150 ± 0.02 MPa of the PGS‐PCL patch. Also, the tensile strength (σ) was 0.100 ± 0.007 MPa, and the maximum elongation (ε) was 92.4 ± 9.2%. For the MagPatch, the *E* was 2.57 ± 0. 27 MPa, σ was 0.374 ± 0.019 MPa, and a maximum elongation was 18.4 ± 2.4%. Both the PGS‐PCL patch and MagPatch showed higher strength in the native myocardium. ^[^ ^23^ ^]^ Cyclic tensile (Figure 1d) and compressive tests (Figure 1e) demonstrated good elasticity and fatigue durability under multiple loading and unloading cycles.

### 2.4 Biocompatibility and Angiogenic Ability of ECs‐SPION

**Figure 2:**
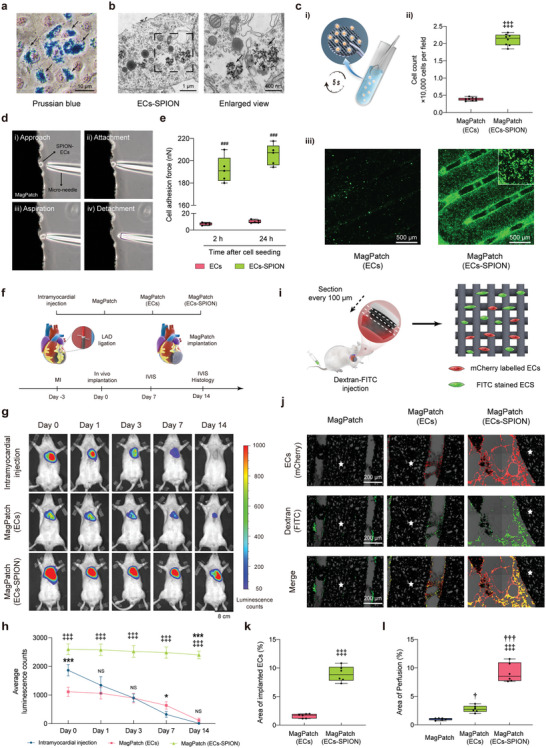
ECs‐SPION were efficiently accumulated in vitro and survived on MagPatch by forming vascular‐like structures connected to the host in vivo. a and b) Representative Prussian staining and TEM images of ECs‐SPION. Black arrows indicate the SPION internalized by ECs. c) MagPatch accumulated ECs‐SPION efficiently via magnetic force in vitro. i) Schematic of the preparation process of MagPatch (ECs‐SPION); ii) Quantification of cells attached to MagPatch with 5 s immersion into cell suspension. (*n* = 8); iii) The representative immunofluorescence images of MagPatch (ECs) and MagPatch (ECs‐SPION) (*n* = 8) d and e) Illustration of micropipette method and measurement of cell adhesive force between MagPatch and cells (*n* = 8). f) Schematic of the experimental design of the 14‐day rat study. g and h) Representative IVIS images measuring the survival of implanted cells 14 days after implantation and quantification of intensity of bioluminescence (*n* = 5). i–l), MagPatch (ECs‐SPION) formed blood network connected to host 14 days after implantation in vivo and quantification of implanted cells and perfusion area (*n* = 6). The white stars indicate patch region. The data were presented as mean ± standard deviation and analyzed using One‐way ANOVA followed by Bonferroni post hoc test. Statistical significance was indicated as follows: ^***^ *p* < 0.001 compared with intramyocardial injection group; ^†††^ *p* < 0.001 and ^†^ *p* < 0.05 compared with the MagPatch group; ^‡‡‡^ *p* < 0.001 and ^‡‡^ *p* < 0.01 compared with the MagPatch (ECs) group. SPION, Superparamagnetic iron nanoparticle; ECs, endothelial cells; TEM, transmission electron microscope.

The isolation of primary ECs was are reported in the Supporting Information. Prussian blue staining was utilized, and the dyed SPION internalized by ECs (**Figure** **2**a). The ferric content was also represented by magnetic resonance imaging and measured by the phenanthroline colorimetric method (Figure S3c, Supporting Information). The results indicated that the labelling ratio and the average internalization of iron per EC increased with increasing concentrations of FluidMAG‐D (Figure S3b,d, Supporting Information). Moreover, the live/dead staining assay showed a similar living cell ratio for the ECs and ECs‐SPION incubated with a concentration of FluidMAG‐D less than 600 µg mL^−1^ (Figure S3e, Supporting Information). A lower living cell ratio was observed after incubation with 800 µg mL^−1^ or more FluidMAG‐D (Figure S3f, Supporting Information). Thus, we selected a FluidMAG‐D concentration of 600 µg mL^−1^ for further experiments, which preserved at least 95% cell viability and 80% labelling efficiency. The transmission electron microscope (TEM) image revealed good internalization of FluidMAG‐D into the ECs at a concentration of 600 µg mL^−1^ (Figure 2b). SPION were encapsulated in endosome‐like structures and distributed in the cytoplasm. The mitochondria in the ECs‐SPION were rod‐shaped without signs of swelling. In addition, EC‐SPION showed an increased angiogenic ability at the transcriptome level compared to ECs (Figure S5, Supporting Information), while the in vitro angiogenic performance showed no significant difference (Figure S4, Supporting Information).

### 2.5 Biocompatibility and Biodegradability of MagPatch

The live/dead staining assay demonstrated a higher ratio of living cells on the PGS‐NdFeB flak compared to PGS‐PCL flak (Figure S6a,b, Supporting Information). The result also showed a higher ratio of living cells on MagPatch compared to PGS‐PCL patch, which can be attributed to the magnetic interaction between the MagPatch and ECs‐SPION (Figure S6c,d, Supporting Information). Immunofluorescence images also showed good attachment of CFs on the surface of the MagPatch (Figure S2g, Supporting Information). The CCK‐8 assay showed that CFs and ECs could grow well on the MagPatch and PCL‐PGS patch with similar cell viability (Figure S2f, Supporting Information). When co‐cultured with primary cardiac myocytes, the cluster beat simultaneously within the MagPatch grid (Movie S4, Supporting Information). Next, an in vitro biodegradation assay of the MagPatch was performed via an enzymatic reaction, and the results indicated that the PGS layer degraded quickly, while the NdFeB layer only degraded slightly (Figure S2d, Supporting Information). The PGS‐PCL composite exhibited significant degradation, with a mass loss of 93.21 ± 0.31% within 3 h. In contrast, the PGS‐NdFeB composite showed minimal mass loss of 3.35 ± 1.17% during the same time period. In addition, one year after the implantation in the heart, the CT value of the MagPatch showed a significant decrease (Figure S11d,e, Supporting Information), indicating the slow biodegradability of the MagPatch in vivo. HE staining further confirmed biodegradation, indicating the complete degradation of the PGS layer and tissue penetration into the NdFeB layer (Figure S11f, Supporting Information). In the hemolytic test, the MagPatch also showed good blood (Figure S2e, Supporting Information) and histocompatibility (Figure S8, Supporting Information).

### 2.6 MagPatch Efficiently Accumulates ECs‐SPION via Magnetic Seeding Technology

The fluorescence microscope images indicated that a large amount of ECs‐SPION could efficiently accumulate onto the surface of the MagPatch, forming a uniform cell patch (Figure 2c; Figure S7a, Supporting Information). The entire process was completed when the MagPatch was immersed in the ECs‐SPION suspension for 5 s, indicating a high cell capture efficiency (Figure S9 and Movie S5, Supporting Information). The representative SEM image also indicated good attachment of ECs‐SPION on the surface of the MagPatch via magnetic force (Figure S7b, Supporting Information). In addition, we further compared the number of attached cells after 5 s and 2 h of immersion of the MagPatch into the ECs‐SPION suspension. The results indicated the similar number of attached ECs‐SPION on the MagPatch, indicating the rapid cellular accumulation via magnetic force generated by MagPatch. The cell adhesion force was measured using the micropipette method. The measurement process consisted of four steps: approach, attachment, aspiration and detachment (Figure 2d). The adhesion force for EC‐SPION seeding was significantly higher than that for EC seeding, and the magnetic force between the MagPatch and a single EC‐SPION was 192 ± 11.64 nN (Figure 2e and Movie S6, Supporting Information).

### 2.7 ECs‐SPION Survive on MagPatch and Form Vascular‐Like Tunnels Connected with the Host Vasculature

The in vitro tube formation assay showed the MagPatch (ECs‐SPION) formed an increased number of nodes and junctions than MagPatch (ECs) group (Figure S9, Supporting Information). The in vivo study design for the MagPatch (ECs‐SPION) is illustrated in Figure 2f. The MagPatch (ECs‐SPION) was sutured on the surface of the infarcted area (Movie S7, Supporting Information), and IVIS was performed to evaluate the survival of the ECs‐SPION. The results indicated that the MagPatch could carry more ECs‐SPION than ECs and that the ECs‐SPION survived 14 days after implantation without a significant decrease (Figure 2j,k). To access vascular‐like structure and evaluate early blood perfusion within MagPatch, rats were anaesthetized and injected intravenously with FITC‐dextran 14 days after implantation. After histologic section (Figure 2g), the results indicated the implanted ECs‐SPION in MagPatch (ECs‐SPION) group were stained with FITC, indicating that the blood network in MagPatch formed vascular‐like structures connected to the host vasculature (Figure 2h,i).

### 2.8 MagPatch (ECs‐SPION) Implantation Improves Cardiac Function and Limits Adverse Left Ventricular (LV) Remodeling after MI

**Figure 3:**
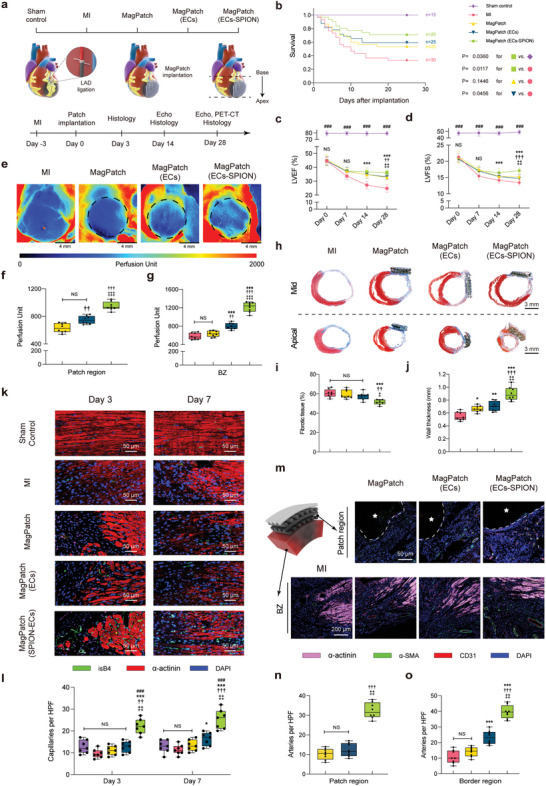
MagPatch (ECs‐SPION) implantation improves blood perfusion and promoted vascular regeneration. a) Schematic of the in vivo experiment design. b) Kaplan–Meier survival analysis after MagPatch implantation. c and d) Echocardiograph data were obtained by echocardiography at days 0, 7, 14 and 28 to measure LVEF and LVFS (*n* = 8). e–g) Representative LSCI images of all groups and quantification of perfusion unit. Black dashed boxes indicate MagPatch region (*n* = 8). h–j) Representative image of Masson's trichrome staining at day 28. Quantitative analysis was conducted to measure the extent of fibrotic tissue and wall thickness. (*n* = 8). k and l) Representative staining in Sham control group and in BZ from MI, MagPatch, MagPatch (ECs) and MagPatch (ECs‐SPION) group for isB4, α‐actinin and DAPI and quantification of the number of capillaries (*n* = 7). m) Representative staining for α‐SMA, CD31, α‐actinin, and DAPI was performed to access small arteries on day 28. The white dashed lines indicated the border of MagPatch and white stars indicated patch region. n and o) Quantification of the number of small arteries in patch region and BZ (*n* = 8). The data were presented as mean ± standard deviation and analyzed using One‐way ANOVA followed by Bonferroni post hoc test. Statistical significance was indicated as follows: ^###^ *p* < 0.001 compared with the Sham control group; ^***^ *p* < 0.001 compared with the MI group; ^†††^ *p* < 0.001, ^††^ *p* < 0.01 and ^†^ *p* < 0.05 compared with the MagPatch group; ^‡‡‡^ *p* < 0.001, ^‡‡^ *p* < 0.01 and ^‡^ *p* < 0.05 compared with the MagPatch (ECs) group. NS indicated *p* > 0.05 compared among groups. MI, myocardial infarction; SPION, Superparamagnetic iron nanoparticle; ECs, endothelial cells; LVEF, left ventricular ejection fraction; LVFS, left ventricular fraction shortening; LSCI, laser speckle contrast imaging; BZ, border zone; HPF, high‐power field.

Echocardiography was performed to assess LV dimensions and cardiac function at multiple time points: baseline (0 and 3 days after LAD ligation), 7 days, 14 days, and 28 days after implantation of the MagPatch (**Figure** **3**a). Parameters such as LV ejection fraction (LVEF) and LV fractional shortening (LVFS) were comparable among all groups at baseline, indicating a stable rat model of MI and similar initial levels of cardiac dysfunction (Figure S17a,b, Supporting Information). After four weeks, the MagPatch (ECs‐SPION) group exhibited a significant increase in LVEF compared to the other groups (Figure 3c). Moderate prevention of LVFS was observed in the MagPatch and MagPatch (ECs) groups, whereas the MagPatch (ECs‐SPION) group showed significant prevention compared to the MI group (Figure 3d). To evaluate the impact of the MagPatch on LV remodeling, viability and necrosis of cardiomyocytes were assessed using PET‐CT (Figure S11a, Supporting Information). The MagPatch (ECs‐SPION) group exhibited a significant reduction in defect size compared with other groups (Figure S11a–c, Supporting Information). Furthermore, Masson's trichrome staining demonstrated a decreased area of fibrotic tissue (Figure 3h,i) and increased LV wall thickness (Figure 3h,j) in the MagPatch (ECs‐SPION) group compared to the other groups.

### 2.9 MagPatch (ECs‐SPION) Promoted Vascular Regeneration and Increased Blood Perfusion

Restoration of the neovascular network is crucial in reestablishing blood and nutrient supply to the ischemic myocardium, which is closely associated with ventricular remodeling. At 28 days post‐implantation, the blood perfusion of the MagPatch was monitored using laser speckle contrast imaging (LSCI) (Figure 3e). The results revealed that the MagPatch (ECs‐SPION) group exhibited higher perfusion in both the patch zone and border zone (BZ) than other groups (Figure 3f,g; Figure S10, Supporting Information), indicating a significant promotion of vascular regeneration. The angiogenic process was further evaluated by quantifying regenerative capillaries and neo‐arteries over time following MagPatch implantation. Capillaries were stained with Isolectin B4 (IsB4), and the results showed a rapid recovery of capillary density in the MagPatch (ECs‐SPION) group on day 3, followed by a persistent increase on day 7 (Figure 3k,l). Additionally, the therapeutic angiogenesis process in the patch zone and BZ was assessed by double staining of heart tissue sections for α‐SMA (vascular smooth muscle cell marker) and CD31 (endothelial cell marker) at 4 weeks post‐implantation (Figure 3m). We also stained α‐actinin to visualize the viable myocardium and distinguish the infarcted tissue from the viable myocardium. The results demonstrated a significantly higher number of α‐SMA‐ and CD31‐positive vascular structures per high‐power field (HPF) in the MagPatch (ECs‐SPION) group in both the patch zone and the BZ than in the other groups (Figure 3n,o).

### 2.10 MagPatch is Effective for NVs‐SPION Accumulation

**Figure 4:**
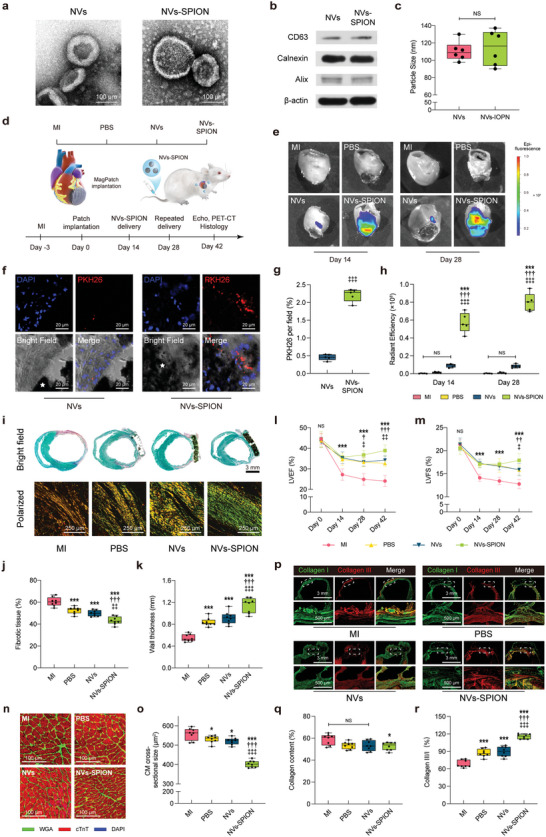
MagPatch accumulated NVs‐SPION and regulated fibrotic process post‐MI. a) Representative TEM image for NVs and NVs‐SPION. b) Representative western blot for surface markers. c) Quantification of particle size of NVs and NVs‐SPION (*n* = 6). d) Schematic of experimental design for 42‐day animal study. e and h) Representative IVIS images and quantification of the fluorescence intensity of isolated hearts (*n* = 5). f and g) The representative immunofluorescence images for NVs and NVs‐SPION in vivo and quantification for area of NVs and NVs‐SPION in vivo. The white stars indicate the area of MagPatch. (*n* = 5). i) Representative images of Picrosirius red staining (top) and polarized light view (bottom) at day 42. j,k) Quantitative analysis of fibrotic tissue and wall thickness at day 42 (n = 8). l,m) LVEF and LVFS were assessed by echocardiography at day 0, 14, 28 and 42. (*n* = 8) n,o) Representative staining with WGA (green), cTnT (red) and DAPI (blue) to visualize cardiomyocytes and quantification of CM cross‐sectional surface area from the BZ (*n* = 8). p) Representative immunofluorescent staining for collagen I (green), collagen III (red) and DAPI. I and j) The ratio of collagen content and collagen III to collagen I among groups (j) (*n* = 5). The data in c, g, h, j, l, m, o, q and r were presented as mean ± standard deviation and analyzed using One‐way ANOVA followed by Bonferroni post hoc test. Statistical significance was indicated as follows: ^***^ *p* < 0.001, ^**^ *p* < 0.01 and ^*^ *p* < 0.05 compared with MI group; ^†††^ *p* < 0.001, ^††^ *p* < 0.01 and ^†^ *p* < 0.05 compared with PBS group; ^‡‡‡^ *p* < 0.001, ^‡‡^ *p* < 0.01 and ^‡^ *p* < 0.05 compared with NVs group. NS indicated *p* > 0.05 compared among groups. MI, myocardial infarction; SPION, Superparamagnetic iron nanoparticle; NVs, nanovesicles; cTnT, cardiac troponin T; WGA, wheat germ agglutinin. BZ, border zone; HPF, high‐power field.

The NVs‐SPION biofabricated by the SPION incubation and size exclusion method had a morphology, diameter and surface marker similar to those of pure NVs (**Figure** **4**a–c). According to the simulation, the flow of NVs‐SPION in the microvessel was simulated to conceptually show the magnetic capture ability of MagPatch (Figure S12, Supporting Information). The in vivo study design for NVs‐SPION is illustrated in Figure 4d. To evaluate the magnetic attraction of the MagPatch, NVs and NVs‐SPION were fluorescently labelled with VivoTrack 680 for the biodistribution assay. The injected NVs‐SPION showed significantly enhanced accumulation in the MagPatch, whereas the pure NVs showed poor accumulation in the heart (Figure 4e,f). In addition, NVs‐SPION were further identified by staining with PKH26 Red. Quantitative analysis of these microscopy images showed that the MagPatch significantly increased the NV‐SPION concentration, which indicated that NVs‐SPION were captured via magnetic force (Figure 4f,g).

### 2.11 NVs‐SPION Captured by MagPatch Improves Cardiac Function and are Effective in Fibrotic Regulation

At 28 days post‐injection of NVs or NVs‐SPION, the NVs‐SPION group exhibited the highest values of LVEF and LVFS values, indicating a further improvement in cardiac function compared to the other groups (Figure 4l,m). The effect of NVs‐SPION captured by MagPatch on fibrotic regulation was also assessed. Collagen fibers were examined using picrosirius red staining and polarization microscopy to determine their content and types at 4 weeks after injection. Type I collagen was identified by yellow staining, whereas type III collagen was identified by green staining under polarized light (Figure 4i). The analysis showed that the total collagen content in the infarction region was comparable among the groups (Figure 4j,k), indicating no significant difference in collagen deposition. However, the NVs‐SPION group exhibited a significantly higher collagen III/collagen I ratio compared to the other groups (Figure 4p–r), indicating the presence of a specific subtype. Furthermore, the cross‐sectional area of the cardiomyocytes in the BZ of the NVs‐SPION group was significantly smaller than that in the other groups (Figure 4n,o).

### 2.12 MagPatch is Effective for Ad‐SPION Accumulation

**Figure 5:**
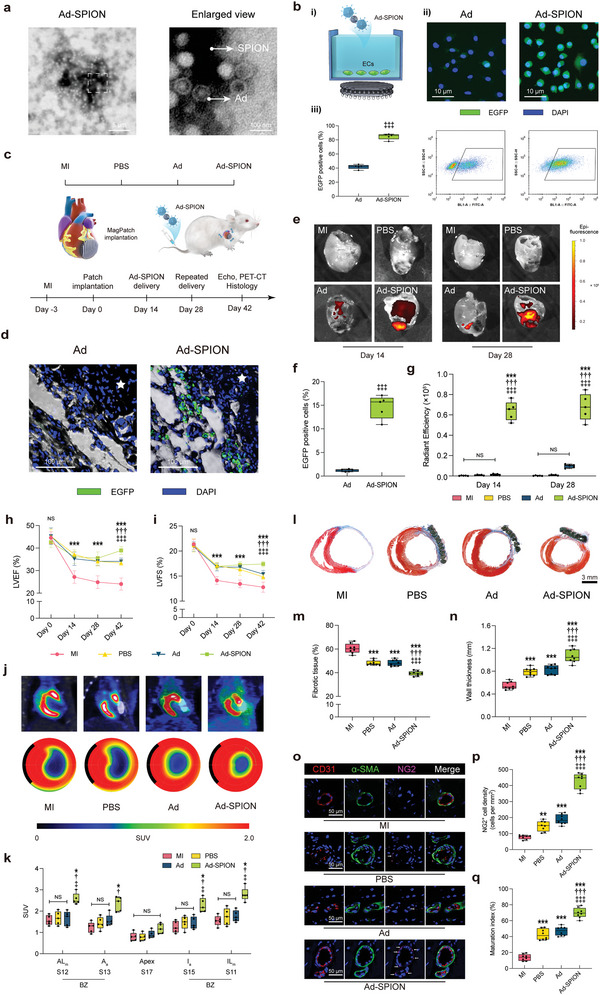
MagPatch accumulated Ad‐SPION and promoted vessel maturation post‐MI. a) Representative TEM image for Ad‐SPION. b) MagPatch enhanced Ad transfection efficiency in vitro. i) Schematic of the transfection; ii) Representative immunofluorescence and flow cytometry images for transfection; iii) Quantification of EGFP positive cells (*n* = 5). c) Schematic of experimental design for 42‐day animal study. d and f) Representative immunofluorescence images for transfection efficiency in vivo and quantification of the number of EGFP positive cells in vivo. The white stars indicate patch region (*n* = 5). e and g) Representative IVIS images and quantification of the fluorescence intensity of isolated hearts (*n* = 5). h and i) LVEF and LVFS were assessed by echocardiography at day 0, 14, 28 and 42 (*n* = 8) j and k) Representative images for ^18^F‐FDG PET and quantification of SUV (*n* = 5). l–n) Representative Masson's trichrome staining and quantitative analysis of fibrotic tissue and wall thickness at day 42 (*n* = 8). o–q) Representative staining for CD31, α‐SMA, NG2 and DAPI and quantification of NG2+ cell density and maturation index. The data were presented as mean ± standard deviation and analyzed using One‐way ANOVA followed by Bonferroni post hoc test. Statistical significance was indicated as follows: ^***^ *p* < 0.001, ^**^ *p* < 0.01 and ^*^ *p* < 0.05 compared with the MI group; ^†††^ *p* < 0.001, ^††^ *p* < 0.01 and ^†^ *p* < 0.05 compared with the PBS group; ^‡‡‡^ *p* < 0.001, ^‡‡^ *p* < 0.01 and ^‡^ *p* < 0.05 compared with the NVs group. MI, myocardial infarction; SPION, Superparamagnetic iron nanoparticle; Ad, adenovirus; TEM, transmission electron microscope; PET‐CT, positron emission tomography‐computerized tomography; SUV, standardized uptake value; BZ, border zone; HPF, high‐power field.

TEM analysis revealed the morphology of Ad‐SPION, showing a close integration of SPIONs with Ad particles (**Figure** **5**a). A schematic representation of the Ad‐SPION transfection procedures is shown in Figure 5b. The infectious capacity and transgene expression of Ad‐SPION in ECs were assessed using flow cytometry and fluorescence microscopy, respectively. Following a 3‐day incubation period, Ad‐SPION, guided by the MagPatch, exhibited significantly higher transfection efficiency than Ad alone, suggesting that the magnetic field could enhance the infection efficiency (Figure 5c). The study design for Ad‐SPION in vivo is depicted in Figure 5d. To examine the biodistribution, Ad and Ad‐SPION were labeled with Cy7, and the results demonstrated a significantly enhanced accumulation of injected Ad‐SPION in the MagPatch, whereas Ad exhibited poor accumulation (Figure 5e–g). Moreover, the transfection efficiency of Ad‐SPION in vivo was quantitatively evaluated by analyzing the number of EGFP‐positive cells in the MagPatch (Figure 5d). These findings indicated the MagPatch increased the transfection efficiency of Ad‐SPION (Figure 5h,i).

### 2.13 Ad‐SPION Captured by MagPatch is Effective in Vascular Maturation and Improves Cardiac Metabolism

Vascular maturation was evaluated by staining for the pericyte marker NG2. Fluorescence images showed pericytes surrounding new vessels in the MagPatch region, indicating stronger vascular maturation in the Ad‐SPION group than in the other groups (Figure 5o). The presence of mature blood vessels facilitates abundant blood supply, improving oxygen and nutrient transport to the ischemic area and enhancing cardiac metabolism. To assess cardiac metabolism after injection, the viability and metabolic intensity of cardiomyocytes were evaluated using PET/CT (Figure 5j). The Ad‐SPION group exhibited a significant increase in standardized uptake value (SUV) compared to the other groups, indicating enhanced metabolic activity (Figure 5k). Furthermore, Masson's trichrome staining revealed a decreased area of fibrotic tissue (Figure 5l,m) and an increased LV wall thickness (Figure 5l,n) in the Ad‐SPION group compared to the other groups, indicating improved tissue remodeling.

### 2.14 The MagPatch is Effective for mRNA‐SPION Accumulation

The in vivo study design for the mRNA‐SPION is illustrated in Figure S15 (Supporting Information). The mRNA‐SPION encoding EGFP were fabricated by co‐incubation of DogtorMag transfection regents and mRNA (Figure S15a,b, Supporting Information). The transfection efficiency of mRNA‐SPION in vivo were further identified via quantitative analysis of EGFP positive cells in MagPatch (Figure S15c, Supporting Information). The results indicated that MagPatch could increase transfection efficiency of mRNA‐SPION (Figure S15d, Supporting Information).

### 2.15 An Orchestrated Design Shielded Excess Magnetic Field and Reduced Off‐Target Effects

The MagPatch was designed with three layers of PGS‐NdFeB on the bottom and one layer of PGS‐PCL on the top. The maximum magnetic field intensity of the top surface was significantly less than that of the bottom surface, indicating the magnetic shielding effect of the MagPatch (Figure S16a, Supporting Information). The Transwell assays showed that the number of migrated cells was significantly decreased with the attenuation of the PGS‐PCL layer (Figure S16b,c, Supporting Information). After implantation onto the epicardium, the MagPatch significantly reduced off‐target effects in the sternum compared to the traditional utilization of an external neodymium magnet as a targeting device (Figure S16d,e, Supporting Information). Moreover, the MagPatch accumulated SPION‐labelled therapeutic agents and reduced off‐target effects in other major organs (Figures S13 and S14, Supporting Information).

### 2.16 The Minimally Invasive Implantation and Magnetic Fixation of MagPatch

The MagPatch can be suturelessly implanted in the epicardium via magnetic force. To fix the MagPatch in the heart, a total volume of 3 mL of alginate hydrogel containing Fe_3_O_4_ nanoparticles was injected directly into the porcine heart at six locations. Then, the MagPatch was then gently placed at the target location and well fitted by the magnetic force between the MagPatch and the hydrogel with Fe_3_O_4_ nanoparticles (Movie S8, Supporting Information).

## 3 Discussion

Our study presents the successful utilization of the MagPatch and ECs‐SPION as a novel approach for vascular cell seeding in engineered circulatory tissue. The MagPatch exhibited efficient accumulation of ECs‐SPION within a short period of 5 s, enabling the rapid formation of a uniform tissue‐engineered patch for immediate implantation. Revascularization is a crucial aspect of cardiac repair following MI, and the timely integration between newly formed vessels and host vasculature is essential for tissue engineering and cardiac regeneration. Furthermore, we propose that revascularization post‐MI can also serve as a vascular route for targeted drug delivery, expanding the therapeutic potential of vascularization beyond its direct effects.

Sufficient elasticity and mechanical strength are critical considerations for epicardial patches. After myocardial infarction, the ventricular wall weakens and experiences increased stress.^[^ ^24^ ^]^ Therefore, myocardial patches with appropriate elasticity and higher strength than the native myocardium are necessary.^[^ ^25^ ^]^ In our study, a four‐layer MagPatch consisting of a PGS‐PCL material as the top layer for shielding the magnetic field and a biocompatible magnetic NdFeB‐blended PGS elastomer as the under layer provided the desired elasticity and mechanical properties. The 3D printing technique employed in fabricating the MagPatch allowed for the creation of a hierarchical microporous structure that supported vascularization and tissue growth. Notably, even without adding cells or drugs, the MagPatch alone exhibits a therapeutic effect.

Compared with conventional approaches utilizing external magnets for cell^[^ ^26^ ^]^ and drug delivery,^[^ ^27^ ^]^ the magnetic cell seeding technology demonstrated in our study offers several advantages. One commonly used approach for vascular reconstruction is spontaneous angiogenesis, which relies on the body's natural healing processes to promote blood vessel formation.^[^ ^28^ ^]^ While spontaneous angiogenesis can occur to some extent in the MI region, it often results in incomplete and inefficient vascularization. Another approach that has been explored is the use of biomaterial‐based scaffolds for tissue engineering and vascular reconstruction.^[^ ^29^ ^]^ These scaffolds provide a structural support for cell attachment and organization, facilitating the formation of blood vessels. However, the integration and survival of implanted cells within these scaffolds can be challenging.^[^ ^30^ ^]^ In our magnetic cell seeding technology, the MagPatch serves as a biocompatible scaffold that not only provides mechanical support but also attracts and accumulates SPION‐labelled ECs. This enables the rapid integration of ECs onto the MagPatch and their subsequent attachment to the host vasculature, resulting in improved vascularization within the MI region. The magnetic cell seeding technology demonstrated in our study offers several advantages for rapid vascular reconstruction and targeted drug delivery in the context of myocardial infarction.^[^ ^28a^ ^]^ The MagPatch enables efficient cell accumulation and uniform tissue formation, promoting rapid integration with the host vasculature. These findings highlight the potential of magnetic cell seeding technology as an innovative approach for tissue engineering and cardiac repair applications. By placing the MagPatch directly in contact with the myocardial infarction area, it generates a magnetic field that attracts cells and drugs without the need for external magnets.^[^ ^27c^ ^]^ Additionally, the two‐layer structure of the MagPatch, with the inside layer providing the magnetic field and the outside layer shielding it, ensures the concentration of cells and drugs specifically on the PGS‐NdFeB side, enhancing the therapeutic efficacy.

Regarding the therapeutic agents, our study employed VEGF‐encoding Ad and NVs derived from bone marrow stromal cells.^[^ ^31^ ^]^ While pro‐angiogenic gene therapy and stem cell therapy have shown promise in preventing heart failure post‐MI, the local retention of these agents in the infarcted myocardium remains a major challenge. Intramyocardial injection, a commonly used delivery method, is limited by its nonrepeatability and the extrusion of injected agents due to continuous muscle contraction. However, intravenous injections exhibit weak effectiveness, poor biodistribution, and low selectivity.^[^ ^14b^ ^]^

Innovative drug delivery systems utilizing micro/nanoparticles have emerged as promising solutions that offer site‐specific and controlled release properties.^[^ ^32^ ^]^ Among these, magnetic‐responsive systems have garnered attention because of their ability to enhance drug accumulation through magnetic force.^[^ ^33^ ^]^ However, previous magnetic‐responsive drug delivery systems have been limited. External magnets and electromagnets have restricted the effective depth and decay of the magnetic force, and their off‐target effects on the thorax are non‐negligible. In addition, maintaining a persistent magnetic field for tissue penetration using externally applied magnets is challenging. The MagPatch overcomes these limitations by enabling direct implantation into the heart and by incorporating a magnetic shielding layer. Our results demonstrate that the MagPatch, under the sustained effect of a magnetic field, enables efficient drug accumulation, facilitating therapeutic treatment at lower doses while reducing adverse effects on healthy organs. By eliminating the need for external magnets and providing a magnetic shielding layer, the MagPatch ensures targeted drug delivery to the myocardial infarction region, reducing off‐target effects and enhancing therapeutic efficacy.

Despite the several promising results, this study still had some limitations. First, the therapeutic effects of the MagPatch were assessed 42 days after implantation, and a longer follow‐up is needed to determine the long‐term therapeutic effects of the MagPatch. Second, the mechanical mismatch between MagPatch and the native cardiac tissue should be noted. The high stiffness of MagPatch might cause mechanical decoupling after in vivo implantation, which could introduce arrhythmia, and many other complications. For the future experiments involving larger animal models, we tend to choose polyurethanes which having a wide range of mechanical properties as basic materials. In addition, we will adjust the printing parameters such as the printing layers and the rotation angle of each layer to adjust the structure. Third, the formation of naive vascular‐like structures rather than more matured vascular structure 14 days post‐MI in the MagPatch (ECs‐SPION) group indicated the potential for further optimization of the MagPatch and ECs. For instance, employing endothelial progenitor cells or adding mesenchymal stem cells could be explored to improve angiogenic ability and promote the maturation of the vascular network. Finally, while the suture less implantation system used in this study demonstrated feasibility for clinical translation, developing a biodegradable implantation system composed of absorbable and biocompatible metals could be an attractive option. These considerations will be important for future studies to address and optimize the limitations of the current study and enhance the clinical potential of magnetic cell seeding technology.

## 4 Conclusion

Taking the design principle of maximum utilization of the magnetic field, we created the MagPatch, which enabled the instant formation of a ready‐made angiogenic organization and rapid anastomosis of the host vasculature after implantation. The prefabricated vascular network within the MagPatch allowed for the efficient accumulation of SPION‐labelled therapeutics, amplifying the therapeutic effects of cardiac repair and reducing off‐target effects. In addition, we designed a magnetic implantation system and suturelessly implanted a MagPatch onto the epicardium using a magnetic force. This study defines an extendable therapeutic platform for vascularization‐based targeted delivery for the treatment of MI and is expected to assist in the progress of regenerative therapies in clinical applications.

## 5 Experimental Section

All animal procedures conducted in this study were approved by the Institutional Animal Care and Use Committee of Charles River Laboratory Animal Technology Co., Ltd, Shanghai, China (Protocol number: P2021097).

All experimental data were expressed as mean ± standard deviation (mean ± s.d.), analyzed using SPSS Statistics (Version 23.0) and plotted using GraphPad Prism 9.2. The exact sample sizes were provided in the figure legends or within the figures themselves. One‐way ANOVA with Bonferroni post hoc test or Student's t‐test was used to determine significant differences. P < 0.05 was considered statistically significant. The detailed statistical information was reported at Statistical Analysis sub‐section in the Supporting Information.

All the experimental details including used reagents, antibodies, and methods were reported in the Supporting Information.

## Conflict of Interest

The authors declare no conflicts of interest.

## Supporting information

Supporting Information

Supplemental Movie 1

Supplemental Movie 2

Supplemental Movie 3

Supplemental Movie 4

Supplemental Movie 5

Supplemental Movie 6

Supplemental Movie 7

Supplemental Movie 8

## Data Availability

The data that support the findings of this study are available in the supplementary material of this article.
