# Supplementary material for: An Intrinsically Magnetic Epicardial Patch for Rapid Vascular Reconstruction and Drug Delivery
Source: Adv Sci (Weinh). 2023 Nov 14;10(36):2303033. doi: 10.1002/advs.202303033 (PMC10754083; doi:10.1002/advs.202303033)
Supplement: Supplementary file 1 — Supporting Information [file ADVS-10-2303033-s007.pdf]

ADVANCED SCIENCE
Open Access

Supporting Information

for *Adv. Sci.*, DOI 10.1002/advs.202303033

An Intrinsically Magnetic Epicardial Patch for Rapid Vascular Reconstruction and Drug Delivery

Bei Qian, Ao Shen, Shixing Huang, Hongpeng Shi, Qiang Long, Yiming Zhong, Zhaoxi Qi, Xiaojun He, Yecen Zhang, Wangxi Hai, Xinming Wang, Yanna Cui, Ziheng Chen, Huixia Xuan, Qiang Zhao, Zhengwei You* and Xiaofeng Ye**

Supporting Information

An intrinsically magnetic epicardial patch for rapid vascular reconstruction and drug delivery

Bei Qian[1], Ao Shen[2], Shixing Huang[1], Hongpeng Shi[1], Qiang Long[1], Yiming Zhong[1], Zhaoxi Qi[1], Xiaojun He[1], Yecen Zhang[1], Wangxi Hai[3], Xinming Wang[1], Yanna Cui[4], Ziheng Chen[5], Huixia Xuan[2], Qiang Zhao[1], Zhengwei You[2]*, Xiaofeng Ye[1]**

B. Qian, S. Huang, H. Shi, Q. Long, Y. Zhong, Z. Qi, X. He, Y. Zhang, X. Wang, Q. Zhao, X. Ye

Department of Cardiac Surgery, Ruijin Hospital, Shanghai Jiaotong University School of Medicine, Shanghai, China.

E-mail: zq11607@rjh.com.cn; xiaofengye@hotmail.com

A. Shen, Z. You

State Key Laboratory for Modification of Chemical Fibers and Polymer Materials, College of Materials Science and Engineering, Institute of Functional Materials, Donghua University, Research Base of Textile Materials for Flexible Electronics and Biomedical Applications (China Textile Engineering Society), Shanghai Engineering Research Center of Nano-Biomaterials and Regenerative Medicine, Shanghai, China.

E-mail: zyou@dhu.edu.cn;

W. Hai

Department of Nuclear Medicine, Ruijin Hospital, Shanghai Jiaotong University School of Medicine, Shanghai, China.

Y. Cui

Department of pharmacology and chemical biology, Shanghai Jiaotong University School of Medicine, Shanghai, China.

Z. Chen

School of Mechatronics Engineering and Automation, Shanghai University, Shanghai, China.

Experimental Section

Materials

Glycerol (analytical grade, 99%), sebacic acid (analytical grade, 99%) and tetrahydrofuran (THF) (anhydrous, ≥99.9%) were purchased from J&K Scientific. Polycaprolactone (PCL) (Mn 80 000 g mol−1) was obtained from Sigma-Aldrich. Salt particulates (sodium chloride, NaCl, 99.5%) were purchased from Shanghai Lingfeng Chemical Reagent Co., Ltd. NdFeB (100 and 400 mesh) were purchased from Magnequench Co., Ltd and grided into 2000 mesh.

Preparation of 3D printing inks

PGS prepolymer was synthesized according to our previously reported method[1]. In brief, equimolar anhydrous glycerol (46 g, 0.5 mol) and recrystallized sebacic acid (101 g, 0.5 mol) were stirred and heated to 135 °C for 24 h with bubbling nitrogen passing through. Then the mixture was stirred in vacuum for another 24 h at the same temperature to obtain a semisolid PGS prepolymer.The PGS-PCL ink was prepared as follows: A THF solution of 9:1 weight ratio PGS prepolymer (1.8 g) and PCL (0.2 g) (20%, w/v) was prepared. 400-600 mesh NaCl particles were ground and sifted. Then, the solution was evenly mixed with NaCl particles (3 g) at a 1:1.5 weight ratio (polymers:NaCl particles) with the assistance of ultrasound. The mixture was placed at 50 °C and 0.5 bar in a vacuum oven for one day to remove the THF. The PGS-NdFeB ink was prepared as follows: A THF solution of PGS prepolymer (2 g) (20%, w/v) was mixed with NaCl particles (3 g) and NdFeB particles (8 g) at a 1:1.5:4 weight ratio (PGS prepolymer: NaCl particles: NdFeB particles). The mixture was placed at 50 °C and 0.5 bar in a vacuum oven for one day to remove the THF.

3D printing of PGS-PCL patches

PGS-PCL patch were 3D printed by FDM similar to our previous method. The PGS-PCL patch was printed by PGS-PCL ink. Both inks were printed at 75 °C (barrel) and 80 °C (nozzel). The parameters of crisscrossed scaffolds were set as follows: The center-to-center distance between filaments was 0.7 mm, with 0°/90° lay-down patterns between two successive layers. The height of every layer was 0.3 mm, and the diameter of nozzle was 0.4 mm. The extrusion rate was 2 μm s^{-1}, and the nozzle move rate was 2 mm s^{-1}. PGS-NdFeB patch were also 3D printed by FDM similar to our previous method. Since the NdFeB powder accounted for 80% by weight in the NdFeB and PGS compound ink, the ink was named magnetic ink-80%.

3D printing of MagPatch

The MagPatch material was 3D printed by FDM by a procedure similar to our previous method[1b]. For each MagPatch, the first 3 layers were printed by PGS-NdFeB ink, while the last layer was printed by PGS-PCL ink. Both inks were printed at 75 ℃ (barrel) and 80 ℃ (nozzle). Then, the printed patches were cured at 100 ℃ for 12 h and 150 ℃ for another 24 h in vacuum. After curing, the patches with salt were put in distilled water and stirred for 24 h to remove the salt. The water was changed every 8 h. Finally, the patches were freeze-dried.

Morphology of patches

The PGS-PCL patch was examined via SEM (SU8010, Jeol, Ltd., Japan) under different magnifications in top view and side view. The morphology and elemental mapping of the MagPatch was examined via SEM (Gemini 300, Zeiss, Germany) with 1 kV voltage and 10 μA current. The patches were beforehand mounted on aluminum stubs and sputtered with gold. A portion of the patches were cutted transversely to reveal the filament cross section.

Mechanical properties

The mechanical properties of the patches were measured by an MTS Echo testing machine (Exceed 40, America). The testing machine was equipped with a 100 N load cell and had a software of TestSuite TW. A thickness gauge was used to measure the sample thickness. In the tensile test, the deformation speed was set as 10 mm min^{-1}. The simples were cut into strips with 4 mm in width and 15 mm in length (n=3). In the single tensile test, the Young's modulus, ultimate tensile strength, and strain at failure of each sample were calculated. In the cyclic tensile test, the sample was stretched to 15 % strain and then loosen to recover to 0 % for 10 cycles. In the cyclic compression test, the samples were cut into 6*6 mm pieces. Each sample was compressed to 60 % strain and released to 0 % at a rate of 1 mm min^{-1} for 10 cycles.

Magnetic properties

The magnetic properties of the NdFeB powder and the MagPatch included a vibrating sample magnetometer (VSM) (7404, Lake Shore Electric, LLC, USA) and Gauss meter (BST200H, Hangzhou BST Magnet, Co., China). The magnetic flux density was analysed by COMSOL Multiphysics® software Version 6.1. Briefly, the "Magnetic Fields, No Currents (MFNC) module" was applied to simulate the spatial and planar distribution of the magnetic field. The digital structure of the three-dimensional MagPatch was designed with four layers, and each layer was perpendicular to the adjusting layers. For the simulation analysis, the width of each

filament was set to 8 mm, and each layer consisted of nine filaments with varying lengths of 2 mm, 4 mm, 6 mm, and 8 mm. The magnetic parameters required for the analysis were obtained through VSM testing of the PCL-PGS and PGS-NdFeB inks.

In vitro degradation of MagPatch

Enzymatic degradation was performed using lipase enzyme (20 000 U g^{-1}, Adamas life) from Candida antarctica in Dulbecco's PBS at an activity of 2000 U mL^{-1}. The patches were cut into small quadrate samples (5*5*1.2 mm). Specimen (n = 4) of 3D-printed PGS-PCL patch and MagPatch were weighed and placed in the aforementioned lipase solution (2 mL per speciman) and incubated at 37 °C. At the predetermined harvest times (30 min, 1 h, 2 h and 3 h), the samples were retrieved, washed with deionized water, and freeze-dried under vacuum. Th degree of degradation was determined by the mass change of the dry weight. SEM (Gemini 300, Zeiss, Germany) was used to observe the degradation process of the patches at 30 min and 2 h at different magnifications.

Primary ECs isolation

Lewis rats (4 weeks) are anesthetized by isoflurane and bled out. The thoracic aorta was fully exposed and removed into a 6 mm dish filled with sterile PBS (4°C). The aorta was cut into 1 mm rings and placed on well coated by solidified Matrigel. Endothelial growth medium was added to cover the aortic segments and the segments were cultured under a moist atmosphere containing 5% CO_2 at 37 °C allowing ECs continue to proliferate for 3 days. Then, the neutral proteinase (50 U/mL) was used for the collection the primary ECs.

ECs-SPION preparation

The ECs used in this research were obtained via isolation and primary culture of Lewis rat aortas. For SPION labelling, the ECs were incubated with FluidMAG-D (Chemicell GmbH) for 24 h with 2 hours of serum starvation prior to particle addition. EC labelling for 24 h with 600 μg of FluidMAG-D per ml of medium was used for the rest of the in vitro and in vivo experiments.

ECs-SPION incubating with different SPION concentration

Magnetic resonance imaging (MRI) experiments were performed via 7-Tesla BioSpec 70/20 scanner (Bruker). The ECs-SPION (5×10^5 cells) incubating with increasing concentrations of FluidMAG-D in 1% agar were filled into tubes and used for T2 measurement. The Prussian

blue and nuclear fast red staining was performed to quantify the labelling efficiency of ECs-SPION. Then the cells were counted and disrupted via ultrasound. The ferric content was measured phenanthroline colorimetric method and the ferric content was calculated.

Characterization of ECs-SPION

For detection of internalized SPION, ECs-SPION were stained with Prussian blue and nuclear fast red. ECs-SPION were cultured in 6-well plate and fixed with 4% paraformaldehyde for 20 min. Prussian blue staining solution was added into cell to incubate for 30 min, and washed with PBS for 3 times. Then, the ECs-SPION was re-dyed with nuclear fast red for 5 min again, and washed with PBS again. After drying, the cell was placed under an optical microscope (Olympus) to observe the blue particles. In addition, submicroscopic structure of ECs-SPION were visualized via TEM. Briefly, ECs-SPION were washed 5 times with PBS and detached with trypsin EDTA. Samples were fixed in 2.5 % phosphate-buffered gluraraldehyde and subsequently post-fixed in a solution of phosphate-buffered 1% osmium tetroxide for just 30 minutes. Images were obtained using ZEISS (GeminiSEM 300) electron microscope operated at 80 kV.

Cell viability of ECs-SPION

For angiogenesis assay, ECs and ECs-SPION (5×10^4 cells/well) were distinguishably seeded on 96-well plates and cultured at 37 °C with 5% CO_2 for 6 hours. CCK-8 assay (Dojindo) was performed according to the manufacturer’s instructions. The optical density (OD) at 450 nm was measured via microplate reader (Thermo Fisher Scientific). The OD ratio of each well was calculated by the following formula: OD ratio = $(OD_{ECs\text{-}SPION} - OD_{blank})/\ (OD_{control} - OD_{blank}) \times 100\%$, where $OD_{ECs\text{-}SPION}$ is the OD value of ECs-SPION, OD_{ECs} is the OD value of ECs and OD_{blank} is the OD value without ECs-SPION and ECs.

Biocompatibility of ECs-SPION

For wound healing assay, ECs and ECs-SPION (2×10^5 cells/well) were seeded on a 12-well plate and incubated at 37 °C with 5% CO_2 to reach confluence. The monolayer was scratched using a tip and washed with PBS. Then the cells were cultured in ECM. ECs and ECs-SPION were visualized and recorded at 0 h, 6 h, 24 h and 48 h post-wounding. The closure area of wound was calculated by the following formula: migration area (%) = $(A_0 – A_1)/A_0 \times 100\%$, where A_0 is the area of initial wound area, A_1 represents the remaining area of wound. For cell cycle analysis, ECs and ECs-SPION were fixed in 4 °C 80% ethanol and stained in Tris-

EDTA buffer containing 40 μg/mL propidium iodide (BD Biosciences) and RNAse A (Sigma). Cell cycle analysis was performed by measuring DNA content via flow cytometer (BD Biosciences). For tumor formation assay, the subcutaneous xenograft nude mouse model was applied to assess the tumor formation ability of ECs-SPION. Briefly, ECs-SPION (3×10^6 cells) resuspended in 100 μl PBS were subcutaneously injected into the left and right axillae of male BALB/c nude mice (18-22g). The ECs were utilized as a control group. Four weeks after injection, the mice were sacrificed and the subcutaneous tissue was harvest for HE staining.

Angiogenic ability of ECs-SPION

For angiogenesis assay, ECs and ECs-SPION were distinguishably seeded in 6-well dishes which coated with 0.5 mm thick Matrigel (4×10^4 cells/cm^2) and allowed to attach for 3 hours. After incubation at 37 °C with 5% CO_2 for 6 h, the plates were washed with PBS. ECs and ECs-SPION cells were imaged under microscope. For later visualization, 4% paraformaldehyde was added to each well and incubate for 15 min at room temperature. The tubular numbers, tubular length and tubular intersecting nodes were counted to evaluate the tube formation ability via Image-Pro Plus software (Media Cybernetics Inc., Bethesda, MD, USA). Each experiment was repeated twice.

qRT-PCR

The total RNA of ECs or ECs-SPION was extracted with TRIzol reagent (Invitrogen) and was then reverse-transcribed to cDNA using qRT-PCR kit (Invitrogen, USA) according to the manufacturer's instruction. qRT–PCR was carried out using the ABI Prism 7300 sequence detection system (Applied Biosystems) and the mRNA level of GAPDH was used as an internal control. Primer sequences are listed in Table S3.

RNA-Seq transcriptome analysis of ECs-SPION

Total RNA was extracted from ECs and ECs-SPION using TRIzol reagent (Invitrogen). The RNA quality was checked using a Bioanalyzer 2200 (Agilent) and the RNA was stored at −80 °C. RNA with an RNA integrity number higher than 6.0 was considered appropriate for experimental cDNA library construction. The sequencing and bioinformatics analyses were performed by Novel Bioinformatics. The differential expression of genes between groups was considered statistically significant when the P-value was <0.05 and the fold change in expression was >1.5. Gene ontology (GO) analysis (http://geneontology.org/) was utilized to

describe the cell functions, including biological processes, molecular functions, and cellular components. A GO term was considered significant with the P-value less than 0.05.

Beating cultures cardiac myocytes on MagPatch

Primary cardiac myocytes were isolated from newborn (3 day-old) Lewis rats as described previously[2]. The cardiac myocytes were intubated with MagPatch after isolation and the beating cultures were visualized and recorded via an optical microscope (Olympus).

Hemolytic assay for MagPatch

For hemolytic test, MagPatch was incubated with rabbit red blood cells for 1 hour at 37 °C. Then the samples were centrifuged and OD of supernatant at 545 nm was measured via microplate reader (Thermo Fisher Scientific). PBS was used as a negative control and sterile water was used as a positive control. The hemolytic activity results were calculated by following formula: Hemolytic ratio = $(OD_{MagPatch} – OD_{negetive})/ (OD_{positive} – OD_{negetive}) \times 100\%$, where $OD_{MagPatch}$ is the OD value after incubation with MagPatch, $OD_{negetive}$ is the OD value of incubation with PBS and $OD_{positive}$ is the OD value of incubation with sterile water. All experiments were repeated three times.

Magnetic assembly of MagPatch (ECs-SPION)

The MagPatch was magnetized using a standard magnetization technique. This involved placing the MagPatch within an electromagnet-generated magnetic field. The electromagnet used in this study consisted of an insulated wire-wrapped ferromagnetic core. By passing an electric current through the wire, a magnetic field was induced within the core, resulting in the magnetization of the MagPatch. To ensure optimal magnetization, we chose to radialize the MagPatch and exposed it to the electromagnet in a radial position. Then the MagPatch was cut into circular discs of 8 mm in diameter and sterilized by ultraviolet light for 3 h for subsequent in vivo study. For the assembly of MagPatch (ECs-SPION), ECs-SPION (5.0×10^6 cells) were suspended in 2 mL PBS, and then the MagPatch was immersed in the EC-SPION suspension for 5 seconds. The process of cell attraction was observed via an optical microscope (Olympus), and the cell accumulation results were also observed via fluorescence microscopy (CKX53, ZEISS). In addition, the microstructure of the MagPatch (ECs-SPION) was visualized via SEM (GeminiSEM 300, ZEISS). As a control group, the MagPatch (ECs) underwent all procedures above except for replacing ECs-SPION with ECs.

Biocompatibility of MagPatch

The magnetized MagPatch was cut into circular discs measuring 4 mm in diameter. The discs were then sterilized using ultraviolet light for a duration of 3 hours to prepare them for biocompatibility testing. CFs derived from Lewis rat hearts were isolated following established protocols[3]. CFs were cultured in high-glucose DMEM supplemented with 10% FBS, 100 U/ml penicillin, and 100 μg/ml streptomycin (Gibco) at 37°C in a 5% CO2 atmosphere. Endothelial cells loaded with superparamagnetic iron oxide nanoparticles (ECs-SPION) were cultured with ECM (ScienCell). The CFs and ECs-SPION were seeded onto the MagPatch at a density of 5.0 × 103 cells per well. After 6 hours of culture to allow cell attachment, the cell-seeded MagPatch discs were transferred to new 96-well plates, and cell viability was assessed using a CCK-8 assay (Dojindo) following the manufacturer's instructions. The optical density (OD) at 450 nm was measured using a microplate reader (Thermo Fisher Scientific). The OD ratio for each well was calculated using the formula: OD ratio = (ODMagPatch − ODblank)/ (ODcontrol − ODblank) × 100%, where ODMagPatch represents the OD value after cell seeding on the MagPatch, ODcontrol is the OD value of ECs seeded on the plate, and ODblank is the OD value without ECs or MagPatch. Live/dead cell staining (ab115347, Abcam) was performed 7 days after seeding the ECs-SPION, and the resulting images were visualized using confocal microscopy (ZEISS).

Measurement of magnetic force between MagPatch and ECs-SPION

To measure the magnetic force between the MagPatch and ECs-SPION, the micropipette aspiration technique was employed, which has been previously used for measuring cell adhesive force [4]. The experimental setup included a pressure control reading device, a micromanipulator (ZEISS), an inverted microscope (ZEISS), and a fabricated micropipette. The micropipette was created from pulled borosilicate glass capillaries with an internal diameter of 5 μm. The critical adhesive force, denoted as Fcritical, was determined using the formula: Fcritical = πr^2ΔPcosθ, where r represents the internal diameter of the micropipette (5 μm), ΔP is the critical vacuum pressure (Pa) required to detach cells from the surface of the MagPatch, and θ denotes the angle between the micropipette and the film surface. For this study, θ was limited to <10°, resulting in cosθ being approximately equal to one. The magnetic force between the MagPatch and ECs-SPION was subsequently calculated using the formula: Fmagnetic = FECs-SPION – FECs, where FECs-SPION represents the adhesive force between the MagPatch and ECs-SPION, and FECs represents the adhesive force between the MagPatch and ECs.

Animal study ethics and in vivo study design

All animal procedures conducted in this study were approved by the Institutional Animal Care and Use Committee of Charles River Laboratory Animal Technology Co., Ltd, Shanghai, China (Protocol P2021097). Adult male Lewis rats weighing between 180-220 g were obtained from Beijing Charles River Laboratory Animal Technology Co., Ltd. The rats were housed in a controlled environment at a temperature of 22 ± 2°C, with a 12-hour light/dark cycle, and provided with standard laboratory chow and sterilized water ad libitum. The in vivo study was divided into two parts. The first part consisted of the following groups: sham control (n=12), MI group (n=25), MagPatch group (n=15), MagPatch (ECs) group (n=12), and MagPatch (ECs-SPION) group (n=12). The second part included the following groups: PBS group (n=15), NVs group (n=8), NVs-SPION group (n=8), Ad group (n=8), and Ad-SPION group (n=8).

Rat model of MI and MagPatch implantation

The rat model of MI was established following previously described methods[5]. Briefly, the rats were anesthetized using 2% isoflurane inhalation and subsequently intubated and ventilated with a respirator (NJKEWBIO, Nanjing, China) set at a respiratory rate of 100 times per minute. A left thoracotomy was performed, and the pericardium was carefully removed. The left anterior descending artery (LAD) was permanently ligated using a 5-0 Prolene suture (Ethicon) to induce the MI model. After ligation, the chest was closed, and the skin was sutured using 3-0 Prolene (Ethicon). Postoperatively, buprenorphine (0.1 mg/kg) was administered twice daily for pain management, and cefazolin (100 mg/kg) was administered twice daily for infection prevention. Echocardiography was performed on the rats at day 0 (2 days after the procedure) to confirm the induction of MI. Subsequently, the rats were anesthetized with 2% isoflurane inhalation, intubated, and ventilated with a respirator. The chest was reopened, and the MagPatch was sutured onto the epicardium of the left ventricle (Movie S7). Following the placement of the MagPatch, the thorax was closed, and the rat was kept on a warm plate. Buprenorphine (0.1 mg/kg) was administered twice daily for postoperative pain relief, and cefazolin (100 mg/kg) was administered twice daily for infection prevention. Animals in the sham group underwent all surgical procedures except for ligation of the LAD artery.

Assessment of ECs-SPION survival on MagPatch

The survival of ECs-SPION on the MagPatch was monitored via IVIS. The ECs were labelled with luciferase by lentiviral transfection prior to SPION incubation. In vivo bioluminescence imaging was performed 0, 1, 3, 7 and 14 days after MagPatch (ECs-SPION) implantation. Intravenous injection of ECs-SPION and MagPatch (ECs) implantation were performed as control groups. To capture the images, rats were anesthetized using 2% isoflurane inhalation and scanned using the IVIS system (Perkin Elmer) 10 minutes after intraperitoneal injection of D-luciferin at a dose of 150 mg/kg. The imaging sequence was acquired using the Spectral Unmixing/Filter Scan tools, with settings including a 1-minute exposure time, excitation filter block, emission filter open, f stops set to 1, field of view set to D, and binning set to 8. The resulting images were analyzed and processed using the Living Image 4.5.5 Software (Perkin Elmer).

Histological assessment of vascular-like structure in MagPatch (ECs-SPION)

ECs were labelled with mCherry by lentiviral transfection prior to SPION incubation to track the ECs-SPION via histological analysis. To access vascular-like structure and evaluate early blood perfusion within MagPatch, rats were anaesthetized with inhaled 2% isoflurane and injected intravenously with FITC-dextran (Sigma) 14 days after implantation. Subsequently, at a 5-minute interval, the rats were euthanized, and their hearts were collected and embedded for subsequent histological analysis.

Assessment of blood perfusion in MagPatch (ECs-SPION)

Laser speckle contrast imaging (LSCI) was utilized to quantitate the blood flow of the MagPatch (ECs-SPION) 28 days after implantation. The rats were anaesthetized, intubated and ventilated with a respirator again. The chest was reopened, and an LSCI device (RWD Life Science) was positioned above the exposed heart. The area of the MagPatch was identified for collection of data, and the perfusion unit was calculated via the manufacturer supplied image software (RWD Life Science).

Assessment of magnetic shielding effect of MagPatch in vivo

To investigate the magnetic shielding effect in vitro, a Transwell assay was conducted. ECs-SPION (1 × 10^5 cells) suspended in 200 µl of ECM were added to Transwell inserts with an 8 µm pore size (Corning). MagPatch discs with different gestures were placed in the lower chamber, while 800 µl of ECM was added to the lower chamber as a chemoattractant. ECs without SPIONs were used as a control. Following 12 hours of incubation at 37 °C in a 5%

CO2 atmosphere, the cells on the Transwell inserts were stained with Calcein AM and Hoechst. The inserts were washed with PBS, and confocal imaging was performed using an LSM880 Meta confocal microscope (ZEISS). Subsequently, the cells were fixed in methanol and stained with 0.5% crystal violet solution at room temperature for 30 minutes. Nonmigrated cells were removed using cotton swabs, and migrated ECs-SPIONs were observed and photographed under an optical microscope (Olympus). The experiment was conducted in triplicate, and the total number of migrated ECs-SPIONs was quantified in five random HPFs. For in vivo investigation of the magnetic shielding effect in vivo, Cy7-labeled SPIONs were visualized on the thorax using an in vivo imaging system (IVIS). MagPatch discs consisting of four layers of NdFeB or one layer of PGS and three layers of NdFeB were sutured onto the surface of the heart. After 14 days of implantation, 20 mg of Cy7-labeled SPIONs suspended in 1 ml of PBS were intravenously injected into the rats. In a separate positive control group, a circular neodymium magnet (1.0 T) was placed on the thorax during and after the injection for 10 minutes. Imaging was performed using the Spectral Unmixing/Filter Scan tools with settings including automatic exposure, excitation/emission at 675/720 nm, f stops set to 2, field of view set to D, and binning set to 8. The acquired images were analyzed and processed using the Living Image 4.5.5 Software (Perkin Elmer).

Biofabrication and characterization of NVs and NVs-SPION

Bone marrow stem cells (BMSCs) from Lewis rats were cultured in NV-free growth media and cocultured with SPIONs (50 nm ferroferric oxide, Sigma) for 48 hours with 2 hours of serum starvation prior to particle addition. BMSC-SPION were thoroughly washed 3 times with PBS to remove extra SPIONs. Then, 2.5×10^8 cells were resuspended in 5 mL iced PBS and sonicated for 5 minutes for cell membrane rupture. The sonicated sample was then sequentially extruded five times through 10 µm, 5 µm, 1 µm, and 400 nm pore membrane filters (Whatman, UK) using a liposome extruder (Avestin, LF-1) to achieve a homogeneous population size. Density-gradient ultracentrifugation was performed by carefully overlaying 10% iodoxanol (Sigma) on top of 50% iodoxanol. The extruded sample was ultracentrifuged at 100,000 × g for 2 hours. The middle layer between the 50% and 10% iodoxanol was collected and subjected to another round of ultracentrifugation. The middle layer was then resuspended in PBS, and NVs-SPIONs were isolated by applying a neodymium magnet at the bottom of the centrifuge tube. Pure NVs isolation underwent all procedures described above except for coculture with SPIONs. The NVs and NVs-SPION were stored at 4°C for immediate use or at −80°C for long-term storage. For electron microscopy visualization, NVs

and NVs-SPION were negatively stained with 2% uranyl acetate solution, and TEM was applied to visualize the complex. Images were obtained using an FEI Tecnai F20 electron microscope. Next, NVs and NVs-SPION were characterized by western blots using anti-CD63, anti-Calnexin and anti-Alix antibodies (Table S3).

Simulation of magnetic targeting for NVs-SPION in MagPatch

COMSOL Multiphysics 6.0 software was utilized to model and analyze the targeting ability of MagPatch for capture of NVs-SPION[6]. The parameters of blood stream were set as the previous described. The inner diameter of blood vessel was 3 μm. The blood density was 1.05 g/cm^3. The consistency index was 1. The power law index was 0.7755. The stress threshold was 0.004 and the critical shear rate was 0.0035. The NVs-SPION was regarded as a paramagnetic particle in the model. The magnetic parameters of NVs-SPION were obtained by the VSM test and the radius of NVs-SPION was obtained by the TEM, setting as 100 nm. The model consisted of a total of 960 particles of NVs-SPION.

Biodistribution of NVs and NVs-SPION in vivo

IVIS was performed to investigate the biodistribution of NVs-SPION and NVs-SPION in vivo. For NVs and NVs-SPION fluorescent labelling, VivoTrack 680 (Perkin Elmer, MA) was applied according to the manufacturer's manual. Next, NVs or NVs-SPION (1 mg, 1.0×10^{11} particles in 200 μl) were intravenously injected into the tail vein of rats with or without MagPatch implantation. The injection was performed 14 and 28 days after MagPatch implantation, and the hearts were harvested 2 hours after injection. The sequence was acquired by the Spectral Unmixing/Filter Scan tools with the following settings: automatic exposure, excitation/emission (675/700), f stops 2, field of view D and binning 8. The specific image was analysed, and the data were processed by Living Image 4.5.4 Software (Perkin Elmer). In addition, NVs and NVs-SPION were stained via PKH26 Red Fluorescent Cell Linker Kits for General Cell Membrane Labelling (Sigma) according to the manufacturer's instructions. After intravenous injection of NVs or NVs-SPION, the rats were euthanized to harvest the hearts and were further sectioned for staining and fluorescence microscope observation. The percentage of PKH26-labelled NVs or NVs-SPION per field area was calculated.

Fabrication of Ad-SPION

Ad encoding EGFP and VEGF164 were synthesized by Shanghai GeneChem Company. The original Ad preparation was stored frozen at -80°C and had a concentration of 1 × 10^10 pfu/ml. To form Ad-SPION, the biotin-streptavidin method was employed using Sulfo-NHS-LC-biotin as the biotinylating reagent, following a previously described protocol[7]. In brief, Sulfo-NHS-LC-biotin was added to 500 µl of Ad solution at a final concentration of 500 ng/ml. The samples were incubated on ice in the dark for 2 hours, and glycine was then used to absorb any unreacted sulfo-NHS-LC-biotin. Ultrafiltration using ZM-500 centrifugal filtration units (Millipore) was performed to remove the unbound biotinylating reagent. Streptavidin-modified SPIONs with an average diameter of 200 nm (Promega) were added to the biotinylated Ad solutions, followed by vortexing for 30 seconds and incubation at room temperature for 30 minutes. The resulting Ad-SPION complexes were stable in aqueous solution and could be stored at -80°C for several months.

Characterization and transduction efficiency of Ad-SPION

For electron microscopy visualization, TEM was applied to visualize Ad-SPION. Images were obtained using an FEI Tecnai electron microscope. For the in vitro transduction assay, ECs were cultured in 48-well plates and incubated with Ad or Ad-SPION in the presence of MagPatch affixed to the bottom of the plate (Figure 5b). After a 48-hour incubation period, the ECs were fixed with 4% paraformaldehyde. They were then subjected to overnight staining with an anti-EGFP antibody (Abcam) at 4°C. Following this, the cells were incubated with FITC-conjugated secondary antibodies (Abcam) for 1 hour at room temperature and counterstained with DAPI. Flow cytometry analysis was performed to determine the percentage of EGFP-positive cells after a 3-day incubation.

Biodistribution of Ad and Ad-SPION in vivo

IVIS was utilized to investigate the biodistribution of Ad and Ad-SPION in vivo. Cy7-PEG-biotin was applied to label streptavidin-modified SPIONs to investigate the biodistribution of Ad-SPION in vivo. Streptavidin-modified SPIONs with an average diameter of 200 nm (Promega) were added to biotinylated Ad and Cy7-PEG-biotin solutions, vortexed for 30 seconds and then incubated at room temperature for 30 mins. Streptavidin-modified Ag nanoparticles were utilized as controls. Ad-encoded EGFP and VEGF164 (1×10^{10} pfu/ml) were intravenously injected into the tail vein of rats with or without MagPatch implantation. The injection was performed 14 and 28 days after MagPatch implantation, and the hearts were harvested 2 hours after injection. The sequence was acquired by the Spectral Unmixing/Filter

Scan tools with the following settings: automatic exposure, excitation/emission (675/720), f stops 2, field of view D and binning 8. The specific image was analysed, and the data were processed by Living Image 4.5.5 Software (Perkin Elmer). In addition, the transfection efficiency was measured via histological analysis. After 7 days of intravenous injection of Ad or Ad-SPION, the rats were euthanized to harvest the hearts and were further sectioned for staining and fluorescence microscope observation.

Fabrication of mRNA-SPION

mRNA encoding EGFP were synthesized by Shanghai Genebiogist. The original mRNA solution was stored frozen at -80°C and had a concentration of 1 μg/μL. DogtorMag transfection regents were utilized to fabricate mRNA-SPION according to manufacturer's instructions. Briefly, we mixed 1 μL of DogtorMag transfection reagent per μg of mRNA and incubate for 5 minutes at room temperature. Then, the mRNA-SPION reagent was diluted using PBS solution for intravenous injection.

Cardiac echocardiography

Cardiac function and structure of the left ventricle (LV) were evaluated using the Vevo 2100 Ultrasound Imaging System (Visual Sonics), following previously described methods[8]. Echocardiographic measurements, including left ventricular ejection fraction (LVEF), fractional shortening (LVFS), left ventricular end-systolic diameter (LVESd), and left ventricular end-diastolic diameter (LVEDd), were collected and analyzed at baseline (day 0, 2 days after ligation), as well as at day 7, day 14, day 28, and day 42 for the respective treatment groups (PBS, NVs, NVs-SPION, Ad, and Ad-SPION). All parameters were determined based on the average of three consecutive cardiac cycles.

^{18}F-FDG PET imaging

Myocardial viability was assessed via the Trans-PET BioCaliburn 700 system (Raycan Technology Co., Ltd., Suzhou, China) as previously described. First, rats were anaesthetized with 2% inhaled isoflurane and 18-fluoro-6-deoxy-glucose (^{18}F-FDG) with an activity of 500±25μCi of was injected through the tail vein. The rats were scanned 1 hour later and images were obtained with the static scanning pattern. Image analysis was conducted in three axes (axial, coronal, and sagittal) using Carimas software (version 2.9) (Turku PET Centre, Turku, Finland). The mean standardized uptake value (SUV) was calculated with the following formula: mean pixel value with the decay-corrected region-of-interest activity

(μCi/kg)/ (injected dose [μCi]/weight [kg]). Infraction size was defined by the fraction of polar map elements with decreased tracer uptake (SUV lower than 50% of the max SUV of the whole heart) in the total polar map.

Histological assessment

The rats were euthanized at specific time points: day 0, day 1, day 3, day 7, day 14, day 28, and day 42. The hearts were carefully collected and perfused with 4% formaldehyde for 20 minutes, followed by rinsing with PBS for approximately 10 minutes. The hearts were then fixed, embedded in paraffin, and sectioned into 5 μm slices on the short axis at 2 mm intervals for subsequent histopathological and morphometric analyses. HE staining, Masson's trichrome staining, and Sirius red-fast green staining were performed. Histological images were scanned using a Pannoramic Digital Slide Scanner (MIDI II, 3D HISTECH, Hungary) to capture images of the patch zone and border zone (BZ). All analyses were carried out by two trained, independent observers who were blinded to the genotype and treatment information. Using Fiji software, various measurements were obtained, including ventricular wall thickness (five random different sites), scar thickness (five random different sites), LV cavity area, and whole LV area[9].

Immunocytochemistry staining

The tissue slices were incubated with specific primary antibodies (Table S1) overnight at 4°C. Subsequently, the slices were incubated with the corresponding secondary antibodies (Table S2) for 1 hour at room temperature. Finally, the slices were counterstained with DAPI. Confocal images were acquired using an LSM880 Meta confocal microscope (ZEISS). To quantify the results, five random fields were selected and counted in each tissue section. For assessment of vascular regeneration, the number of capillaries per HPF was defined by the signal of isB4 while the artery per HPF was defined by signal of CD31 and α-SMA. Capillaries were defined as dotted, liner or circledis B4 signal. Capillary density was assessed histomorphometrically by manual delineation of capillaries in five high-power fields at BZ, and a mean was established. Care was taken to exclude arterioles and large vessels (with diameter more than 10 μm) when counting capillaries.

Magnetic implantation of the MagPatch

Sutureless implantation of the MagPatch discs was facilitated by using an alginate hydrogel integrated with Fe3O4 nanoparticles. Fe3O4 nanoparticles, with an average diameter of 50

nm, were incorporated into a sodium alginate solution (2% wt/vol), while a calcium alginate suspension (1.5% wt/vol) was employed for crosslinking. Osmotic pressure was controlled by the addition of mannitol. A total volume of 3 mL of the hydrogel was directly injected into the porcine heart at six designated locations, ensuring a slow withdrawal of the needle to prevent any potential leakage. Subsequently, the MagPatch was carefully positioned over the injection site and secured onto the epicardium through the magnetic force between the MagPatch and the Fe3O4 hydrogel.

Statistical analysis

The data were represented as bar plots displaying the mean value accompanied by the standard deviation (s.d.), or as box-and-whisker plots indicating the median value (represented by a black bar inside the box) and the minimum and maximum values (represented by the bottom and top whiskers, respectively). Statistical diagram was plotted using GraphPad Prism 9.1. The exact sample sizes (n values) for each experiment were provided in the figure legends or within the figures themselves. Statistical analysis was performed using a two-tailed Student's t-test to compare two groups, while a one-way ANOVA with Bonferroni post hoc test was employed for comparisons involving more than two groups. Kaplan-Meier curves were used to depict cumulative survival, and differences in cumulative survival were assessed using the log-rank test in IBM SPSS Statistics (Version 23.0). A p-value of less than 0.05 was considered statistically significant.

Supplementary Figures

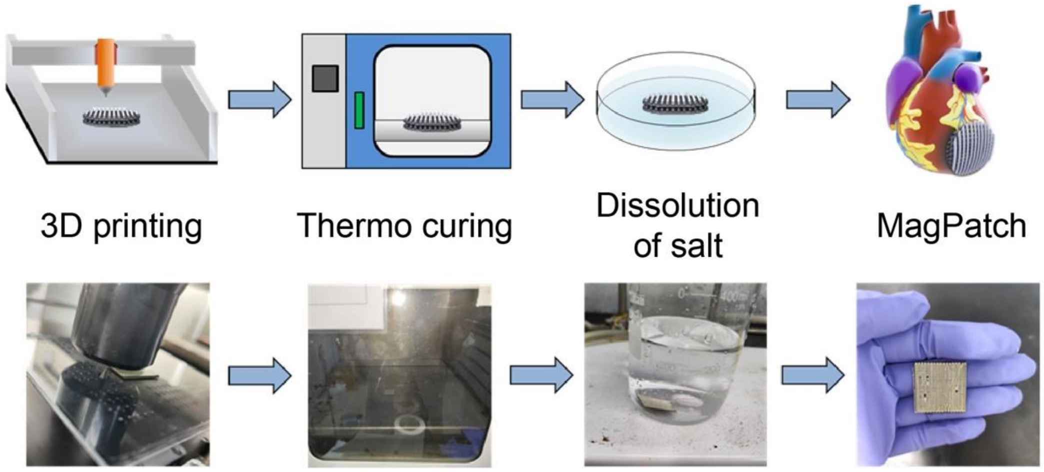

Figure S1. Schematic representation for fabrication of MagPatch.

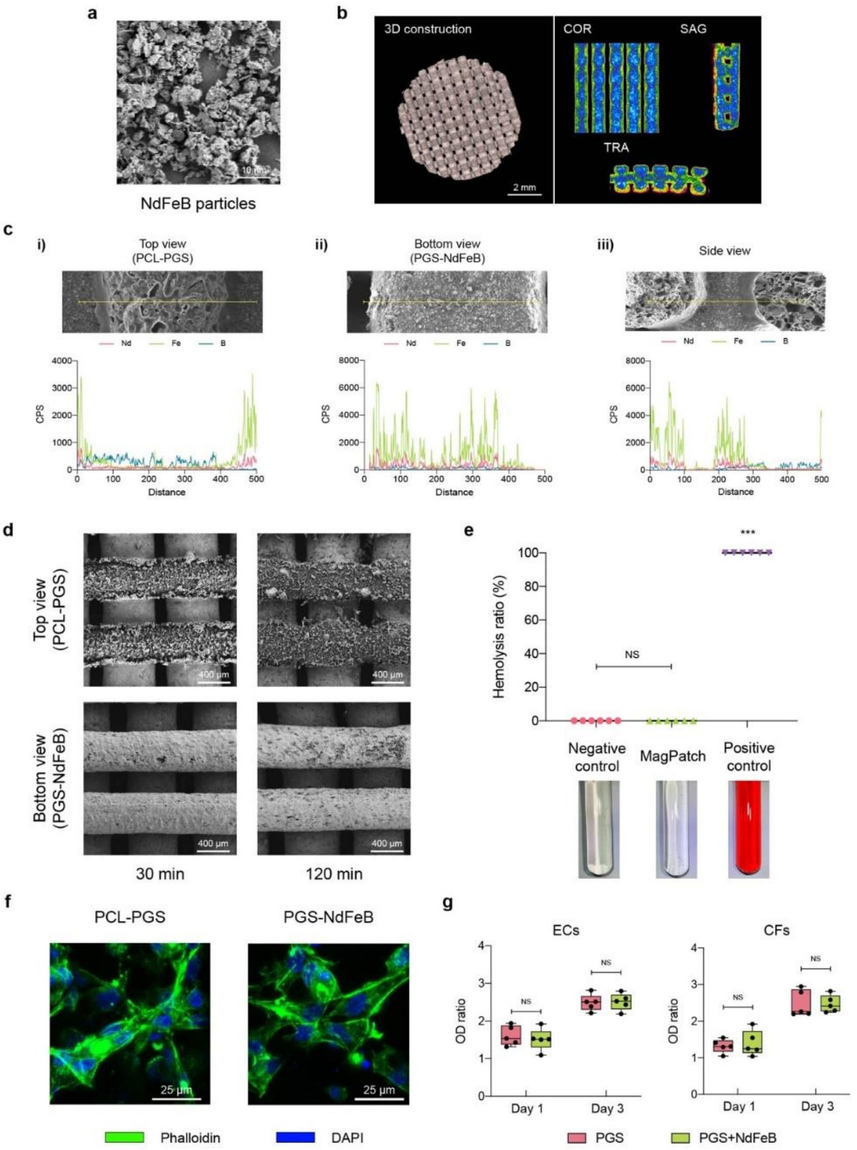

Figure S2. The composition and in vitro biocompatibility of MagPatch. a, Representative SEM image of NdFeB particles utilized for MagPatch. **b**, Micro-CT scanning of MagPatch in three axes including SAG, TRA and COR and representative 3D constructive image of MagPatch. **c**, The representative SEM-EDS images of MagPatch. (i) The line scanning image of top view indicating the main components of PCL-PGS. (ii) The line scanning image of bottom view indicating the main components of PGS-NdFeB. (iii) The line scanning image of side view. **d**, Representative SEM images of MagPatch after 30 minutes and 120 minutes incubation with lipase for in vitro degradation experiment. **e**, Quantitative analysis of hemolysis ratio (top) and representative photos of hemolytic assay (bottom) (n = 6). The PBS was utilized as negative control while sterile water was utilized as positive control. **f**, Representative staining for phalloidin to assess cytoskeleton of CFs seeding on the surface of PCL-PGS and PGS-NdFeB. **g**, Quantification of ECs and CFs viability by CCK-8 assay on day 1 and 3 (n = 5). The data were expressed as mean ± standard deviation and analyzed using

One-way ANOVA followed by Bonferroni post hoc test. ***p<0.001 compared with negative control group. SPION, superparamagnetic iron nanoparticle; ECs, endothelial cells; SEM, scanning electron microscope SAG, sagittal view; TRA, transverse view; COR, coronary view; Micro CT: micro computed tomography; EDS, energy disperse spectroscopy; ECs, endothelial cells; CFs, cardiac fibroblasts.

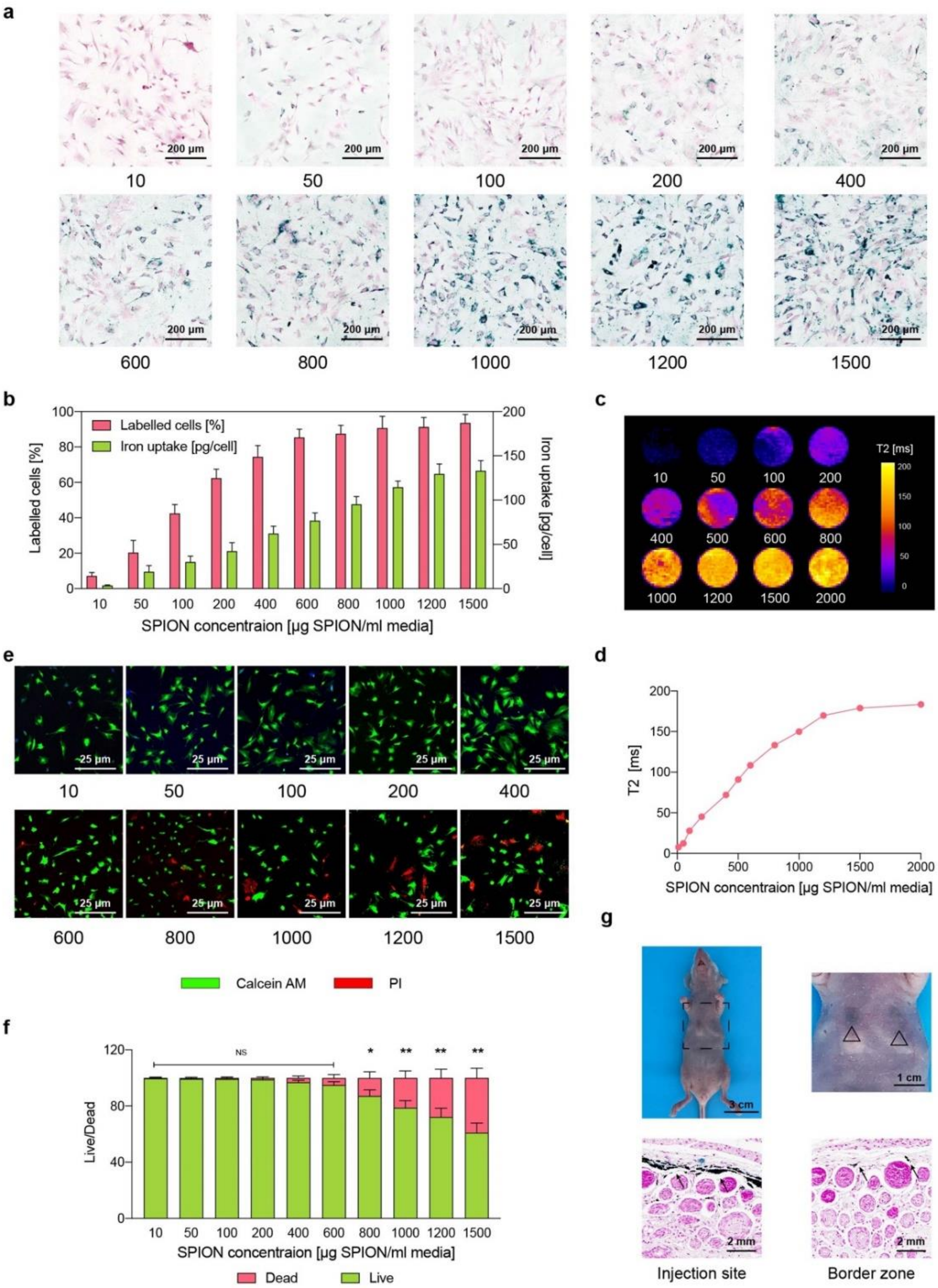

Figure S3. In vitro biocompatibility of ECs-SPION. a, Representative Prussian blue and nuclear fast red staining images of ECs after 24 hours incubation with different SPION concentration. **b**, Quantitative analysis of labelled cells and iron uptake of ECs after 24 hours

incubation with different SPION concentration (n = 5). **c**, **d**, Representative magnetic resonance T2 contrast map of tubes filled with agar containing ECs after 24 hours incubation with different SPION concentration (**c**) and quantitative analysis of T2 value for labelled ECs-SPION (**d**) (n = 3). **e**, **f**, Representative Live/Dead staining for Calcein AM and PI to assess biocompatibility of ECs after 24 hours incubation with different SPION concentration (n = 3) and quantitative analysis of live and dead rate (n = 3). **g**, Representative gross observation after 28 days ECs injection for tumor formation assay and representative HE staining image of subcutaneous tissue indicating no sign of tumorigenesis. The black triangles indicated the injection site and the black arrows indicated the injected SPION in the subcutaneous tissue. The data were expressed as mean ± standard deviation and analyzed using One-way ANOVA followed by Bonferroni post hoc test. $^{**}p<0.01$ and $^{*}p<0.05$ compared with ECs group. SPION, superparamagnetic iron nanoparticle; ECs, endothelial cells.

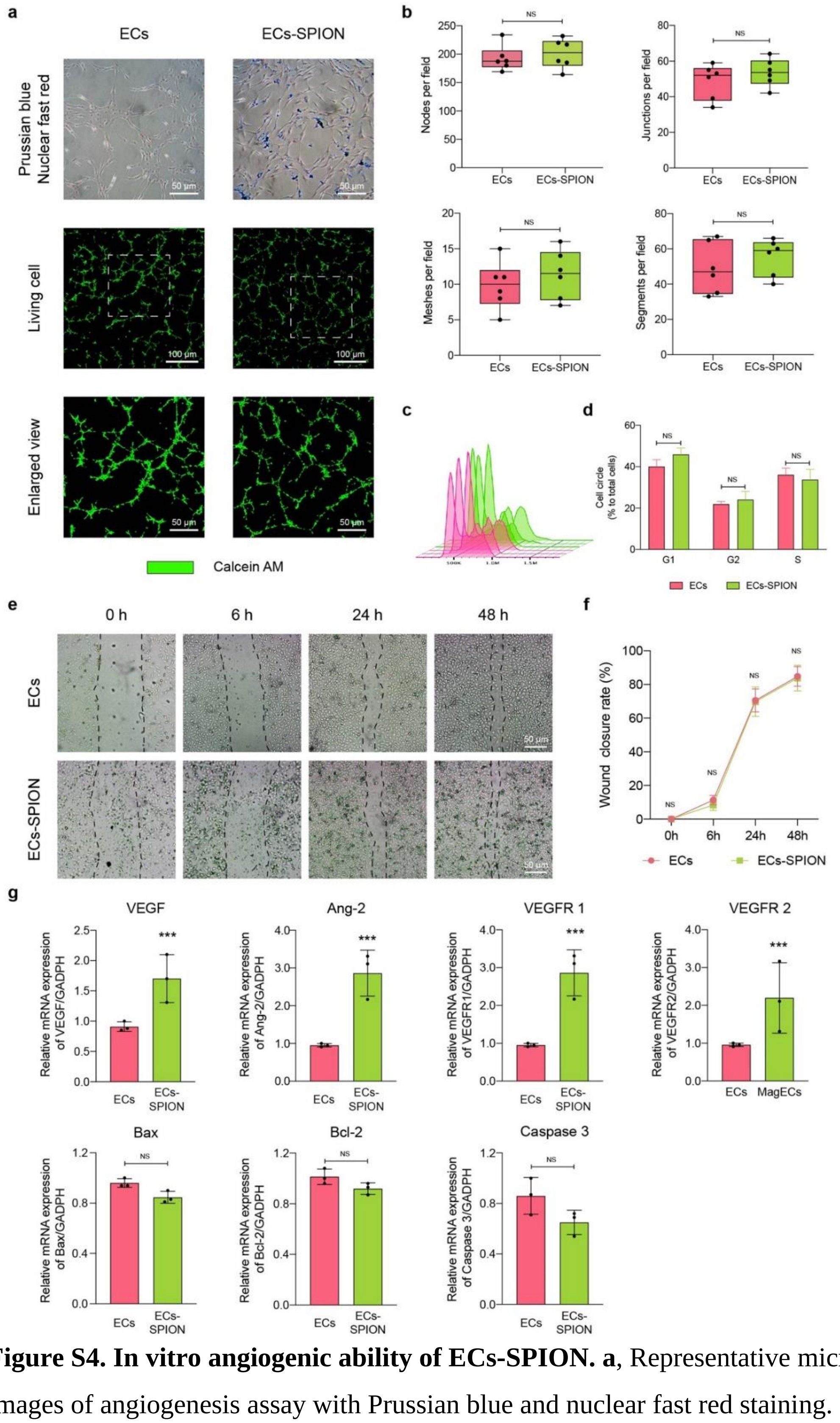

Figure S4. In vitro angiogenic ability of ECs-SPION. a, Representative microscopic images of angiogenesis assay with Prussian blue and nuclear fast red staining. Representative staining for Calcein AM to assess angiogenic ability of ECs and ECs-SPION. **b**, Quantitative analysis of the number of nodes, junctions, meshes, segments formed by ECs and ECs-SPION (n = 6). **c**, **d**, Representative cell cycle images measured by flow cytometry (**c**) and

quantitative analysis of the percentage of cells at each cell stage (**d**) (n = 3). **e**, **f**, Representative microscopic images of in vitro wound healing assay for ECs and ECs-SPION (**e**) and quantitative analysis wound closure rate (**f**) (n = 3). **g**, Quantitative analysis of mRNA expression related to angiogenesis and apoptosis by qPCR (n = 3). The data were expressed as mean ± standard deviation and analyzed using One-way ANOVA followed by Bonferroni post hoc test. $^{***}p<0.001$ compared with ECs group. SPION, superparamagnetic iron nanoparticle; ECs, endothelial cells.

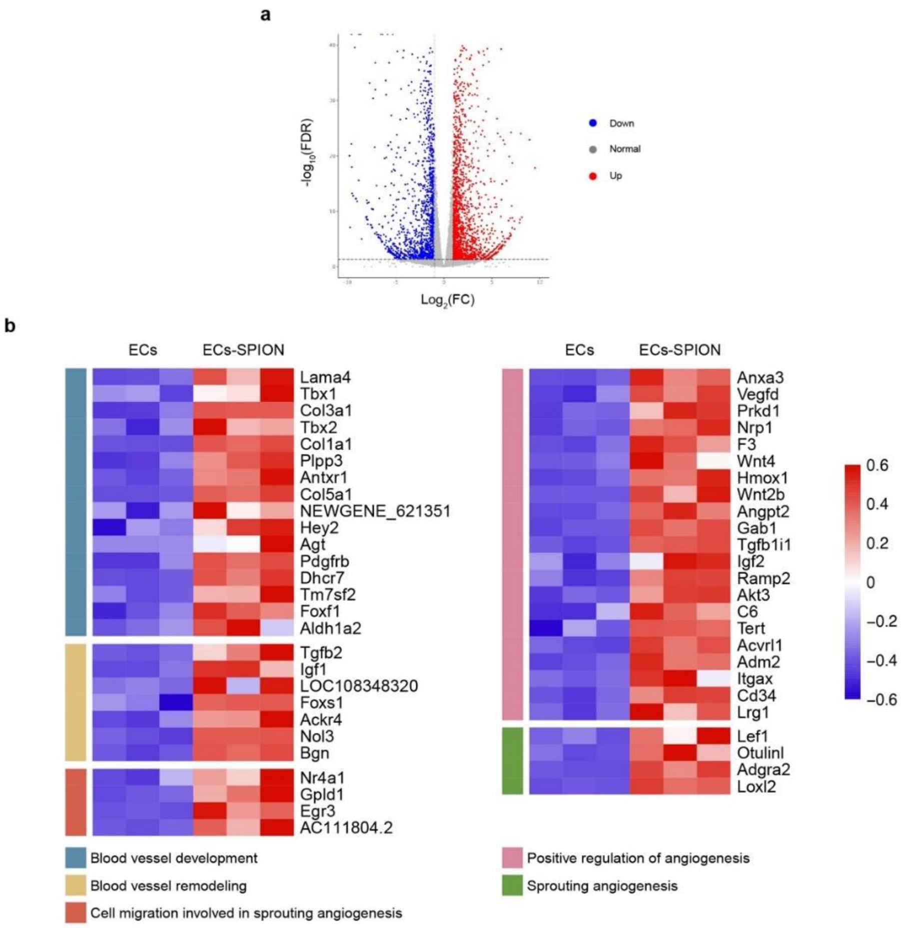

Figure S5. ECs-SPION showed an increased angiogenic ability from transcriptome level. **a**, Expression of the genes between ECs group and ECs-SPION group (n = 3). **b**, The heatmap of angiogenesis-related genes expression between ECs group and ECs-SPION group. SPION, superparamagnetic iron nanoparticle; ECs, endothelial cells.

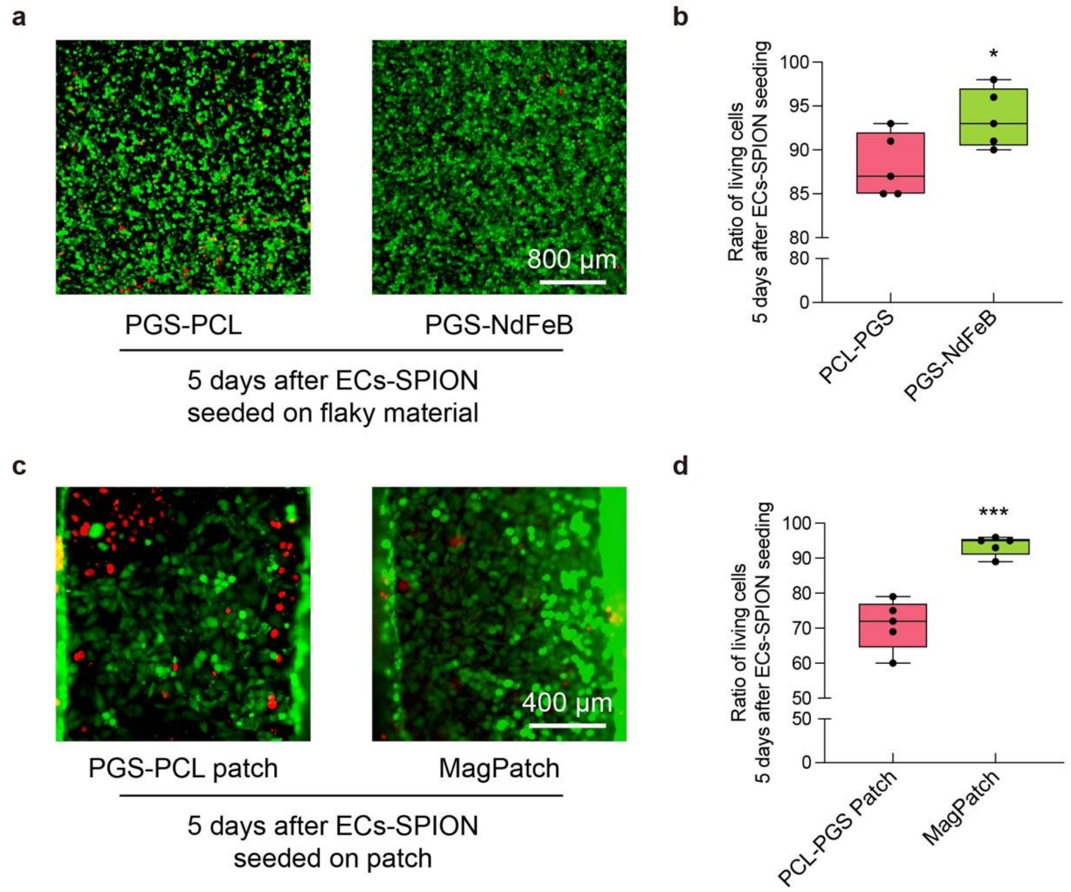

Figure S6. MagPatch exhibit good biocompatibility in vitro. a, Representative fluorescent microscopy images of live/dead staining for ECs-SPION seeded on PGS-PCL flak or PGS-NdFeB flak after 5 days incubation. **b**, Quantitative analysis of living ECs-SPION on PGS-PCL flak or PGS-NdFeB flak after 5 days incubation (n = 5). **c**, Representative fluorescent microscopy images of live/dead staining for ECs-SPION seeded on the PGS-PCL patch or PGS-NdFeB patch after 5 days incubation. **b**, Quantitative analysis of living ECs-SPION on PGS-PCL patch or PGS-NdFeB fpatch after 5 days incubation (n = 5). The data were expressed as mean ± standard deviation and analyzed using t-test. * $p<0.05$ and ***$p<0.001$ compared with PGS-PCL group. SPION, superparamagnetic iron nanoparticle; ECs, endothelial cells.

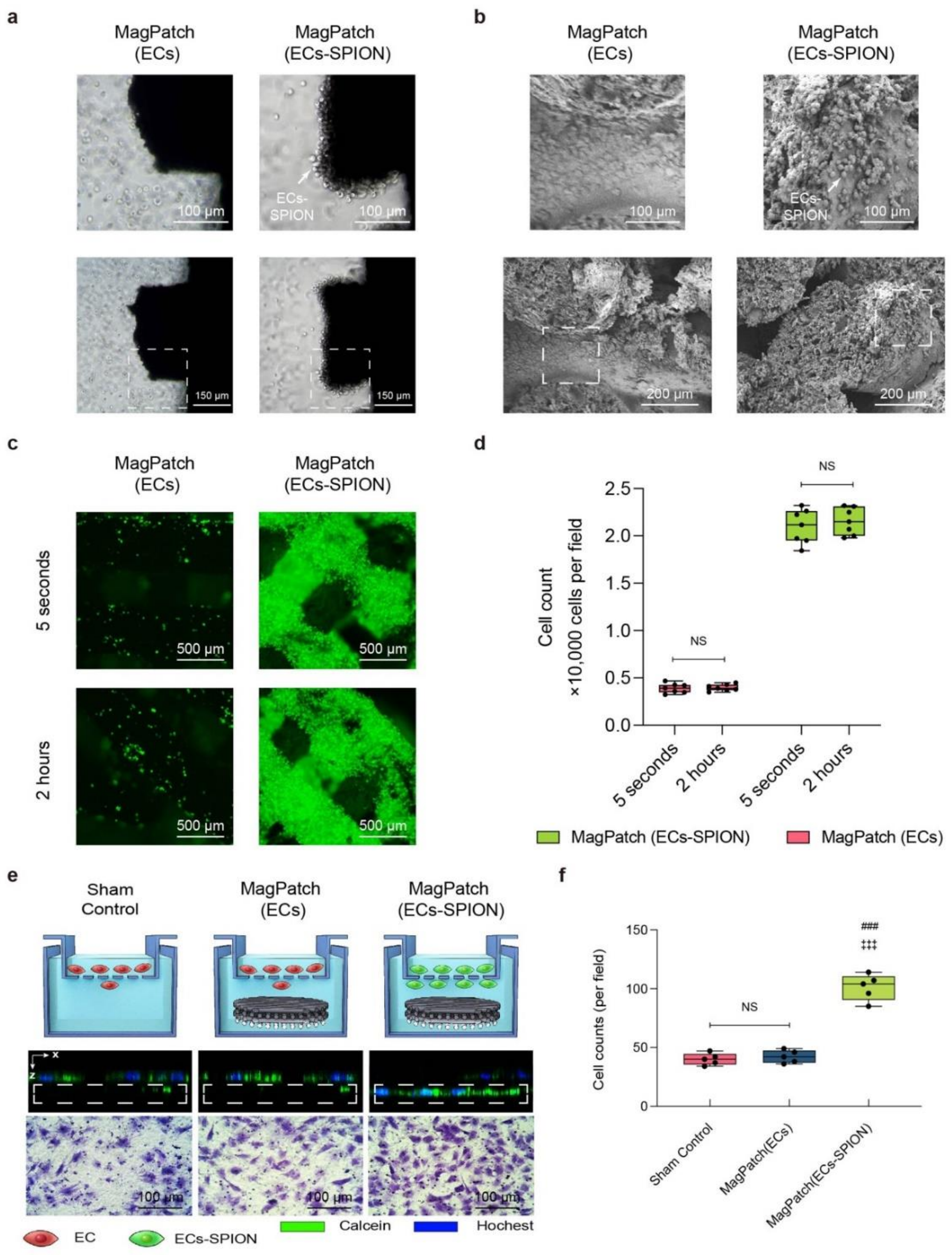

Figure S7. MagPatch efficiently enrich ECs-SPION via magnetic force in vitro. a, Representative photograph after MagPatch immersed in ECs suspension (left) and ECs-SPION suspension (right). The white arrow indicated the enriched ECs-SPION. **b**, Representative SEM image after MagPatch immersed ECs suspension (left) and ECs-SPION suspension (right) for 5 seconds. The white arrow indicated the enriched ECs-SPION. **c**, Representative fluorescent microscopy images of enriched ECs-SPION on MagPatch after 5 seconds or 2 hours incubation in vitro. **d**, Quantitative analysis of enriched ECs-SPION on the MagPatch after 5 seconds or 2 hours incubation in vitro (n = 7). **e**, Schematic of Transwell assay for assessment of magnetic enrichment ability of MagPatch (top). Representative confocal microscopy images (middle) and crystal violet staining images (bottom) of migratory

cells indicated the MagPatch could enrich ECs-SPION efficiently in vitro. **f**, Quantitative analysis of migrated cells on the porous membranes of Transwell inserts among the three groups (n = 5). The data in **d** and **f** are expressed as mean ± standard deviation and analyzed using One-way ANOVA followed by Bonferroni's post hoc test. $^{\#\#\#}$p<0.001 compared with Sham control group; ‡‡‡p<0.001 compared with MagPatch (ECs) group. SPION, superparamagnetic iron nanoparticle; ECs, endothelial cells; SEM, scanning electron microscope.

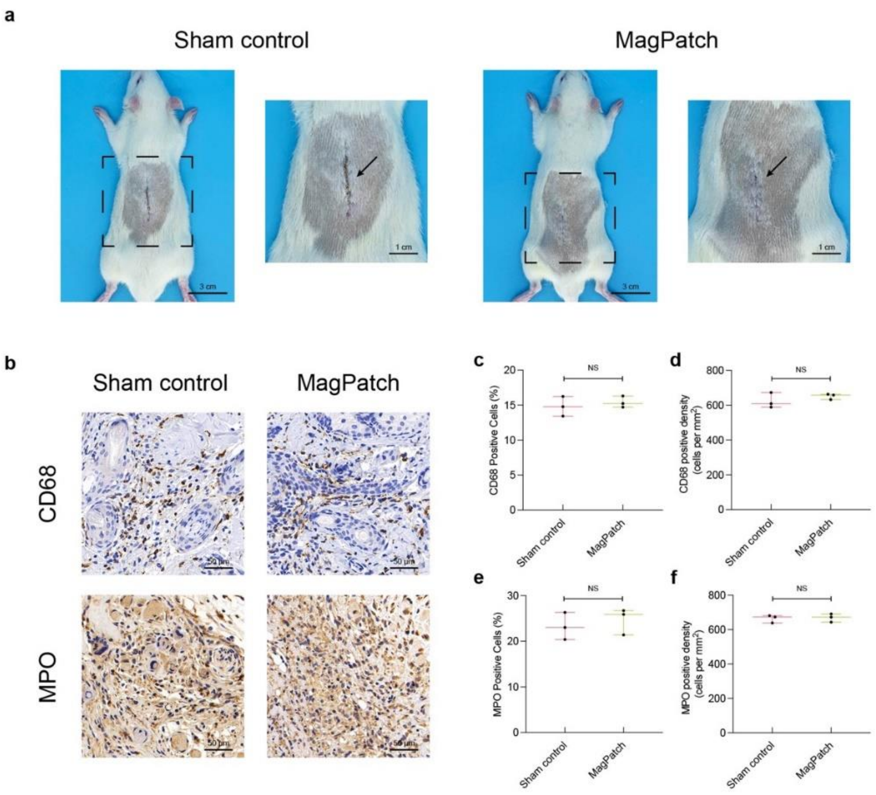

Figure S8. Inflammation response after subcutaneous implantation of MagPatch. a, Representative photograph after sham operation and subcutaneous implantation of MagPatch at day 7. The black arrows indicated the surgical incisions. **b**, Representative immunohistochemical image for CD68 (top) and MPO staining. **c**, **d**, Quantitative analysis of CD68 positive cells (**c**) and positive areas (**d**) (n = 3). **e**, **f**, Quantitative analysis of MPO positive cells (**e**) and positive areas (**f**) (n = 3). The data were expressed as mean ± standard deviation and analyzed using t-test. SPION, superparamagnetic iron nanoparticle; ECs, endothelial cells.

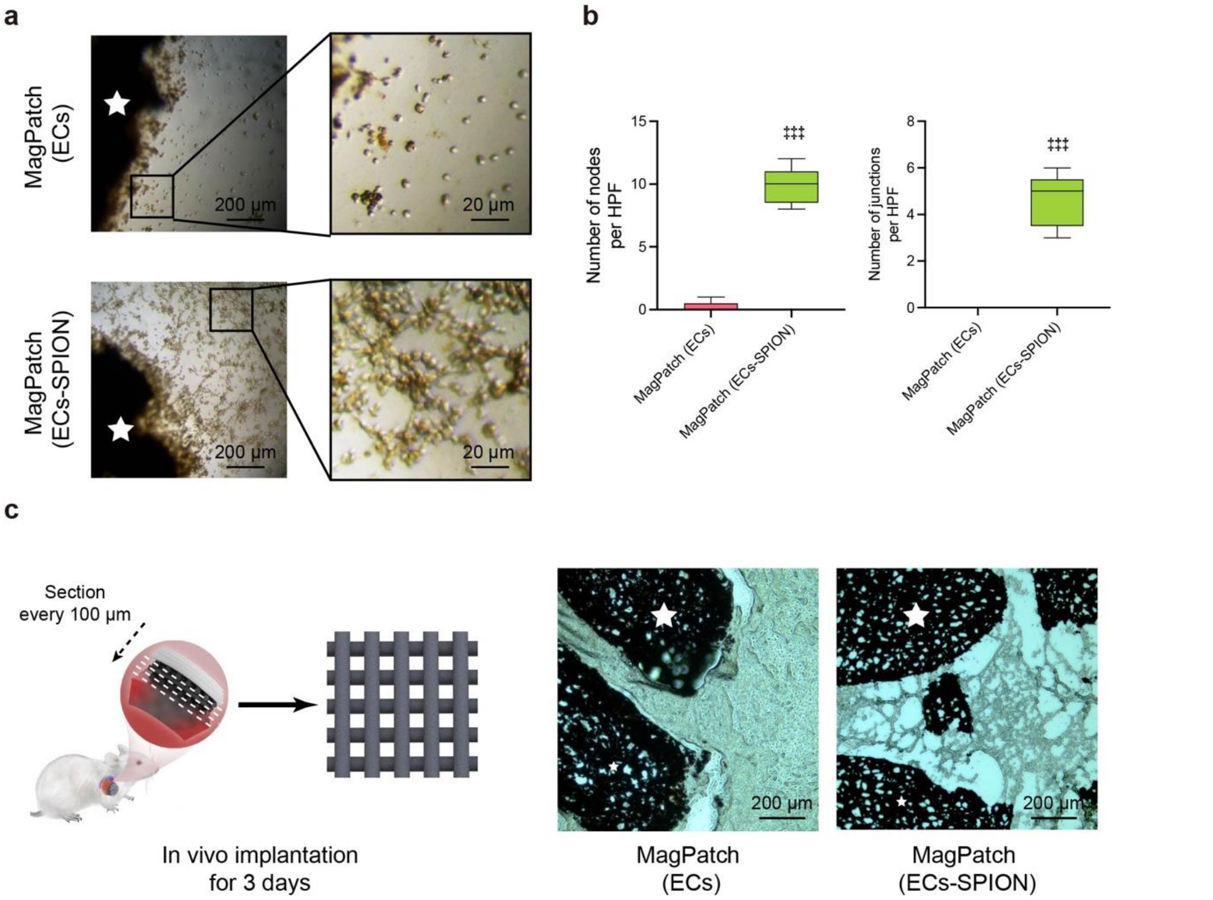

Figure S9. MagPatch (ECs-SPION) could form vascular-like structure in vitro and in vivo. a, Representative photograph of tube formation assay for ECs or ECs-SPION on the MagPatch after 5 days incubation. The white star indicated the MagPatch area. **b**, Quantitative analysis of nodes and junctions formed by ECs or ECs-SPION on the MagPatch after 5 days incubation (n = 5). **c**, Representative light microscopy images of MagPatch after 3 days implantation upon heart in vivo. The white star indicated the MagPatch area. The data were expressed as mean ± standard deviation and analyzed using t-test. ‡‡‡p<0.001 compared with MagPatch (ECs) group. SPION, superparamagnetic iron nanoparticle; ECs, endothelial cells.

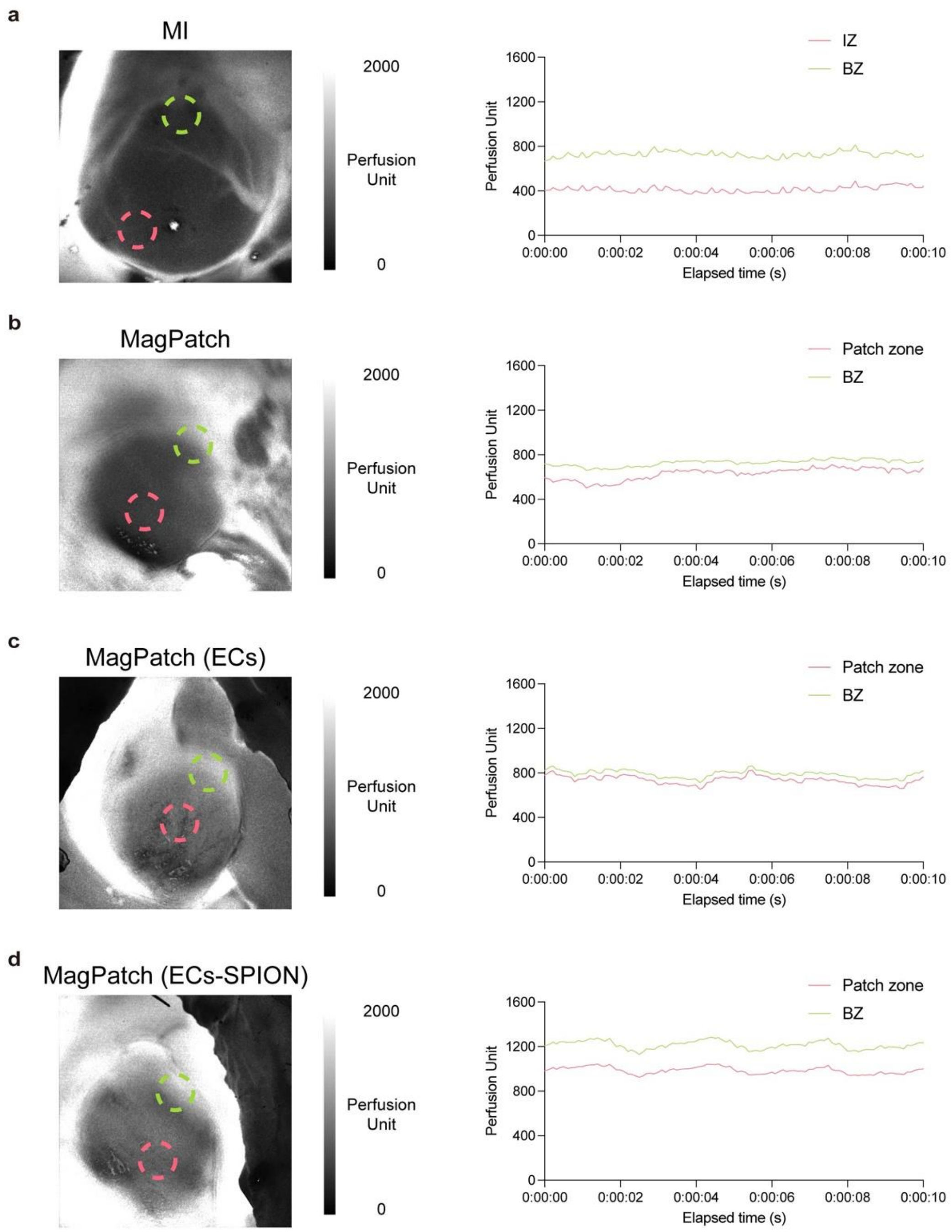

Figure S10. Assessment of the perfusion unit in patch region and BZ via LSCI for MI, MagPatch, MagPatch (ECs) and MagPatch (ECs-SPION) group. a, Representative LSCI images of MI and quantification of perfusion unit in patch region and BZ. The pink dashed cycle indicated the IZ and green dashed cycle indicated the BZ. **b-d**, The representative LSCI images of MagPatch, MagPatch (ECs) and MagPatch (ECs-SPION) group and quantification of perfusion unit in patch region and BZ. The pink dashed cycle indicated the patch region and green dashed cycle indicated the BZ. MI, myocardial infarction; SPION, superparamagnetic iron nanoparticle; ECs, endothelial cells; LSCI, laser speckle contrast imaging; BZ, border zone.

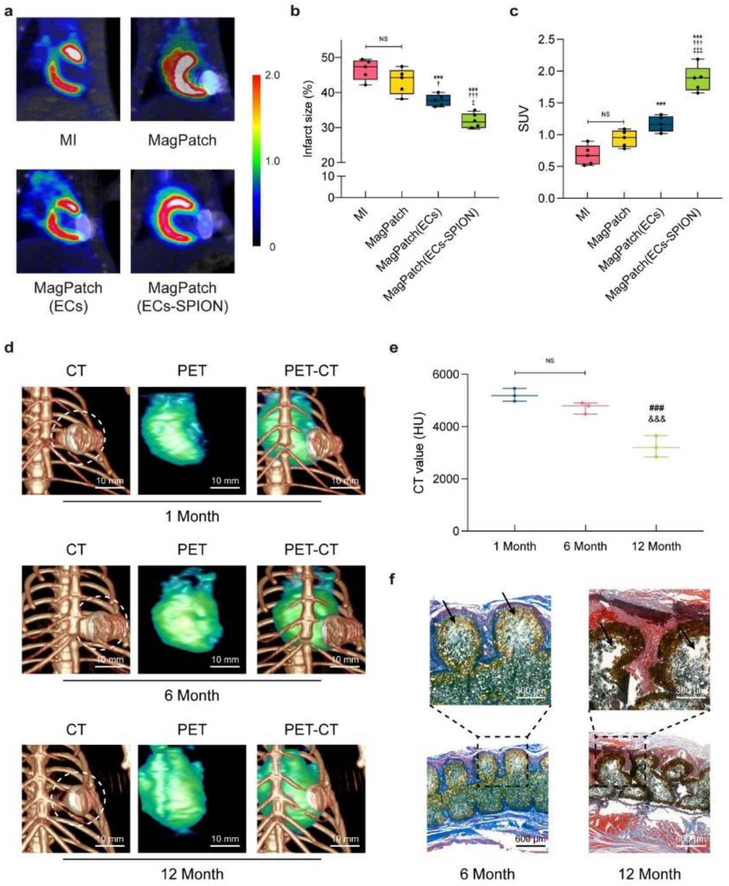

Figure S11. MagPatch (ECs-SPION) promoted cardiac metabolism and exhibited slow degradable process in vivo. a, Representative images of PET-CT with ^{18}F-FDG at day 28 and assessment of cardiomyocyte metabolism by SUV. **b**, **c**, Quantitative analysis of cardiomyocyte metabolism by SUV (**b**) and quantitative assessment of infarcted size (**c**) at day 28 (n = 5 independent animals per group). **d**, Representative images of PET-CT with ^{18}F-FDG after 1-, 6- and 12-month post MagPatch (ECs-SPION) implantation. **e**, Quantitative analysis of CT value after 1-, 6- and 12-month post MagPatch (ECs-SPION) implantation (n = 3), indicating slow degeneration of MagPatch in vivo. **d**, Representative images of Masson staining after 6- and 12-month post MagPatch (ECs-SPION) implantation. The black arrows indicated the degraded region of MagPatch (ECs-SPION). The data were expressed as mean ± standard deviation and analyzed using One-way ANOVA followed by Bonferroni's post hoc test. $^{***}p<0.001$ compared with MI group; $^{\dagger\dagger\dagger}p<0.001$ and $^{\dagger}p<0.05$ compared with MagPatch group; $^{\ddagger\ddagger\ddagger}p<0.001$ and $^{\ddagger}p<0.05$ compared with MagPatch (ECs) group; $^{\#\#\#}p<0.001$ compared with 1 month after MagPatch (ECs-SPION) implantation; $^{\&\&\&}p<0.001$ compared with 6 months after MagPatch (ECs-SPION) implantation. SPION, superparamagnetic iron nanoparticle; ECs, endothelial cells; SUV, standard uptake value.

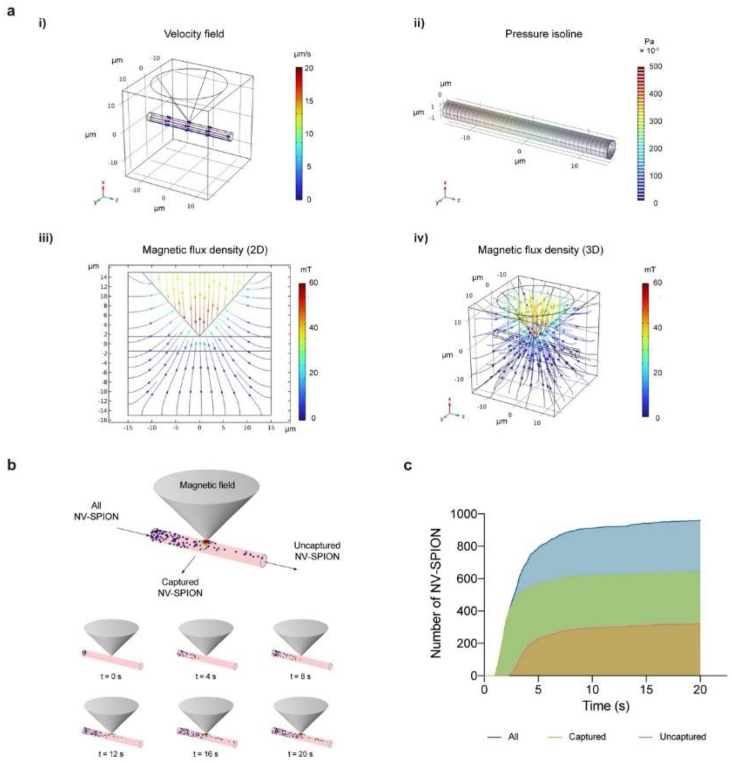

Figure S12. Simulative capture of NVs-SPION by MagPatch. a, Computer simulation of blood vessel and magnetic field generated by MagPatch. (i) The simulated velocity field of blood vessel; (ii) Simulated pressure isoline of blood vessel; (iii) The simulated plane magnetic field distribution of MagPatch; (iv) The simulated spatial magnetic field distribution of MagPatch. **b**, Flowing simulation of NVs-SPION in blood vessel under the guidance of MagPatch (top) and representative images of NVs-SPION capture at different time point (bottom). **c**, Quantitative analysis of captured rate of NVs-SPION under the guidance of MagPatch. NVs, nanovesicles; SPION, superparamagnetic iron nanoparticle.

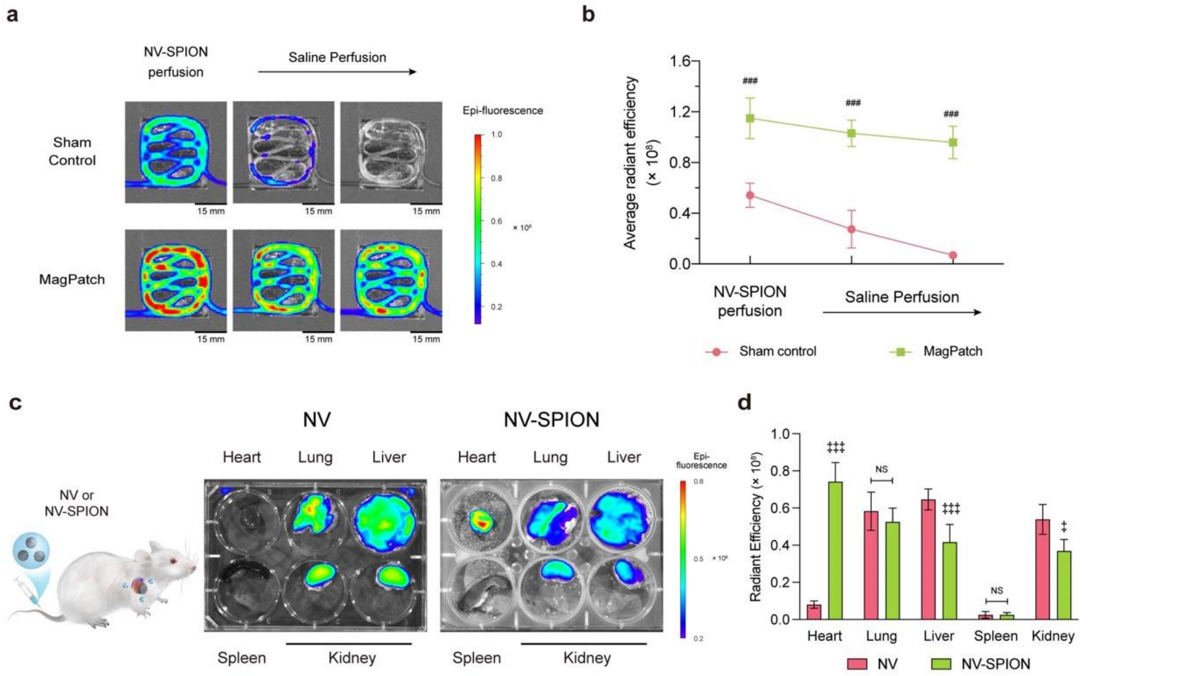

Figure S13. MagPatch accumulated NVs-SPION via magnetic force and reduced off-target effects in other organs. a, Representative IVIS images of captured NVs-SPION under the guidance of MagPatch, indicating the MagPatch could capture NVs-SPION efficiently in vitro. **b**, Quantitative analysis of radiant efficiency generated by captured NVs-SPION (n = 4). **c**, Schematic of NVs or NVs-SPION injection after 42 days post MagPatch implantation. Representative IVIS images measuring the off-target effect in lung, liver, spleen and kidney. **d**, Quantification of the fluorescence intensity of isolated organs (n = 3). The data in **b** and **d** are expressed as mean ± standard deviation and analyzed using t-test. $^{###}p<0.001$ compared with Sham control group; $^{‡‡‡}p<0.001$ and $^{‡}p<0.05$ compared with NVs group.

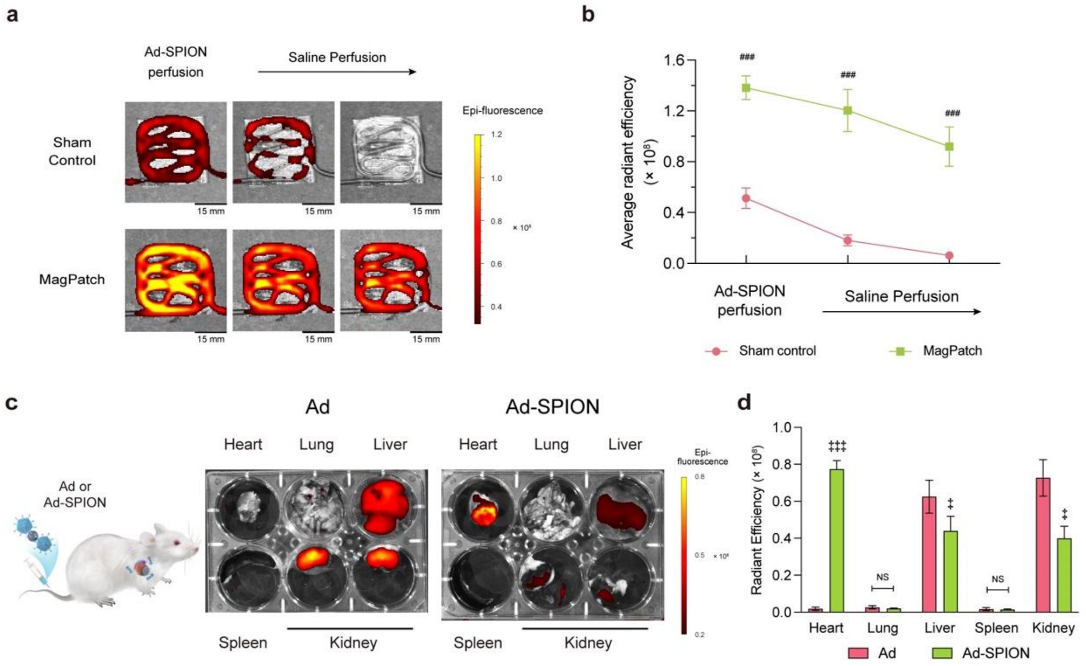

Figure S14. MagPatch accumulated Ad-SPION via magnetic force and reduced off-target effects in other organs. a, Representative IVIS images of captured Ad-SPION under the guidance of MagPatch, indicating the MagPatch could capture Ad-SPION efficiently in vitro. **b**, Quantitative analysis of radiant efficiency generated by captured NVs-SPION (n = 4). **c**, Schematic of Ad or Ad-SPION injection after 42 days post MagPatch implantation. Representative IVIS images measuring the off-target effect in lung, liver, spleen and kidney. **d**, Quantification of the fluorescence intensity of isolated organs (n = 3). The data were expressed as mean ± standard deviation and analyzed using t-test. $^{###}p<0.001$ compared with Sham control group; $^{‡‡‡}p<0.001$ and $^{‡}p<0.05$ compared with Ad group.

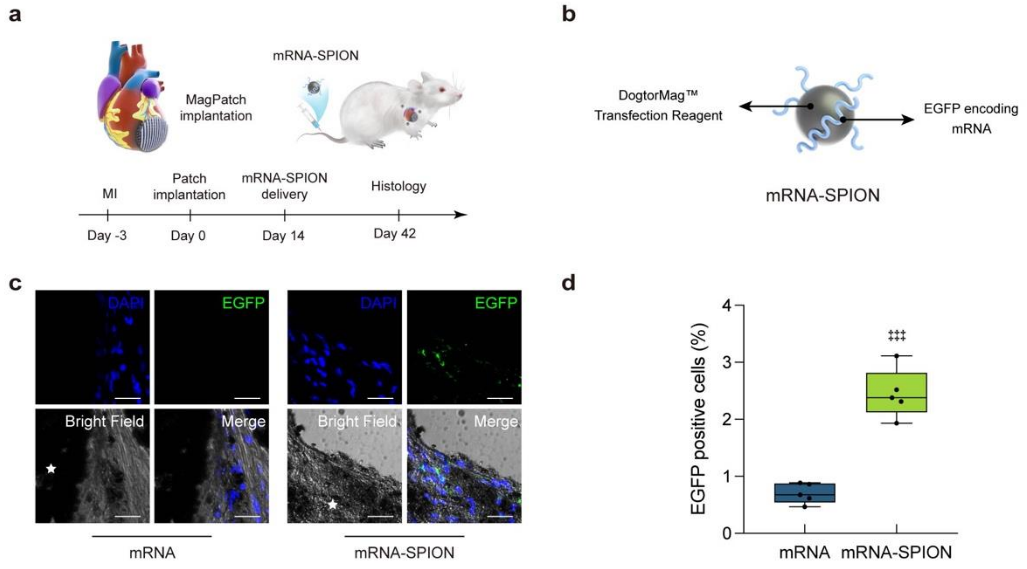

Figure S15. MagPatch accumulated mRNA-SPION in vivo. a, Schematic of experimental design for 42-day animal study. **b**, Schematic diagram of mRNA-SPION. **c**, **d**, The representative immunofluorescence images for transfection efficiency of mRNA and mRNA-SPION in vivo (**c**) and quantification of the number of EGFP positive cells in vivo (**d**). The white stars indicate the area of MagPatch (n = 5). The data were expressed as mean ± standard deviation and analyzed using t-test. ‡‡‡p<0.001 compared with mRNA group.

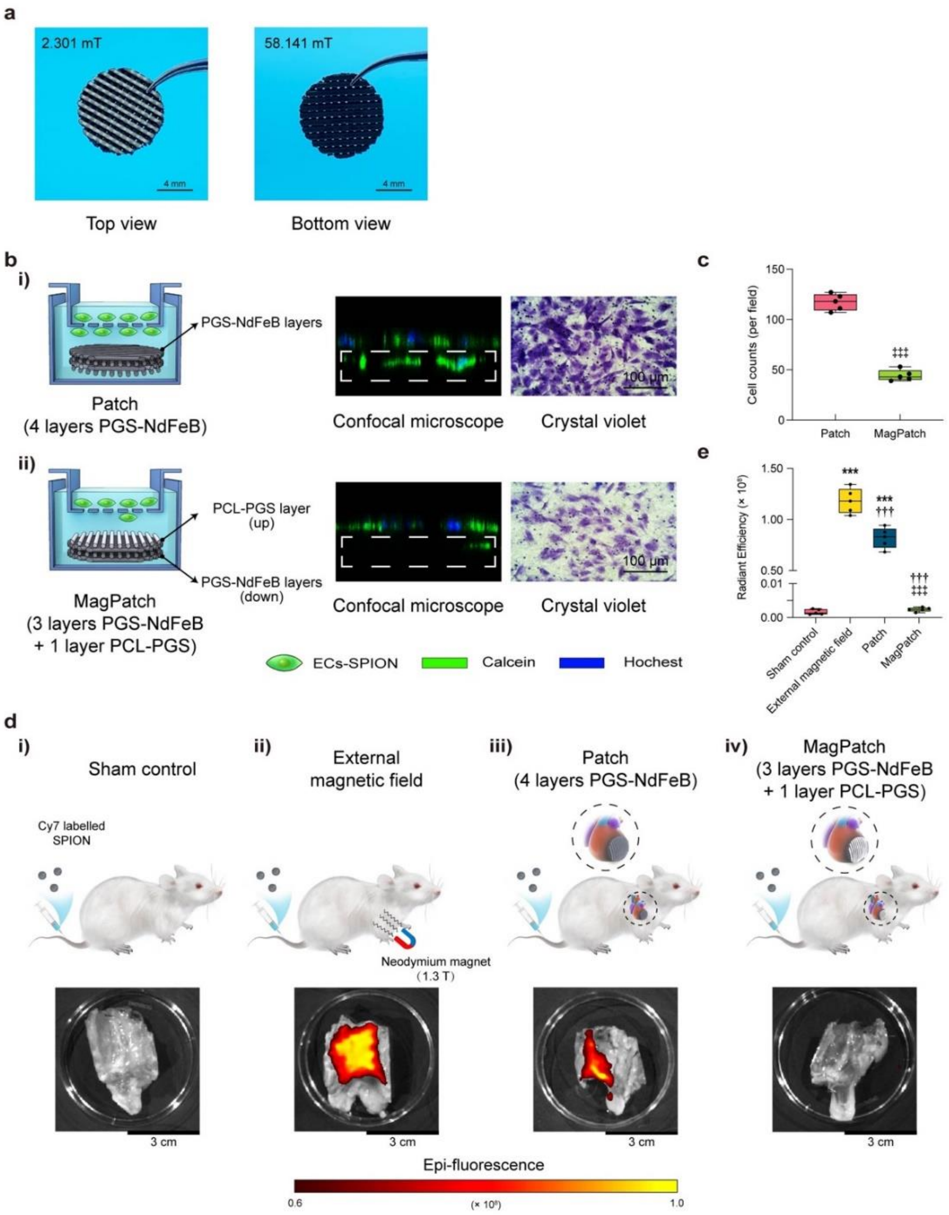

Figure S16. MagPatch shielded the excess magnetic field and reduced off-target effects in sternum. a, Maximum magnetic field intensity of top (left) and bottom (right) surface. **b**, Schematic of Transwell assay for in vitro assessment of magnetic shielding effect of MagPatch (left). Representative confocal microscopy images (middle) and crystal violet staining images (right) of migratory cells. (i) Patch group: the patch contained 4 layers of

PGS-NdFeB; (ii) MagPatch group: the MagPatch contained 3 layers of PGS-NdFeB (down) and 1 layer of PCL-PGS (up). **c**, Quantitative analysis of migrated cells on the porous membranes of Transwell inserts among the three groups (n = 5). **d**, Schematic of in vivo assessment of magnetic shielding effect of MagPatch (up) and representative IVIS images (down) measuring the off-target effect of sternum. (i) Sham control group: injection of Cy7-labelled SPION only; (ii) External magnetic field: injection of Cy7-labelled SPION with a neodymium magnet (1.3 T) placed on the chest; (iii) Patch group: injection of Cy7-labelled SPION after 28 days post patch (4 layers of PGS-NdFeB) implantation; (iv) Patch group: injection of Cy7-labelled SPION after 28 days post MagPatch (3 layers of PGS-NdFeB and 1 layer of PCL-PGS) implantation. **e**, Quantification of the fluorescence intensity of isolated sternum (n = 3). The data were expressed as mean ± standard deviation and analyzed using One-way ANOVA followed by Bonferroni's post hoc test. $^{***}p<0.001$ compared with Sham control group; $^{\dagger\dagger\dagger}p<0.001$ compared with External magnetic field group; $^{\ddagger\ddagger\ddagger}p<0.001$ compared with Patch group.

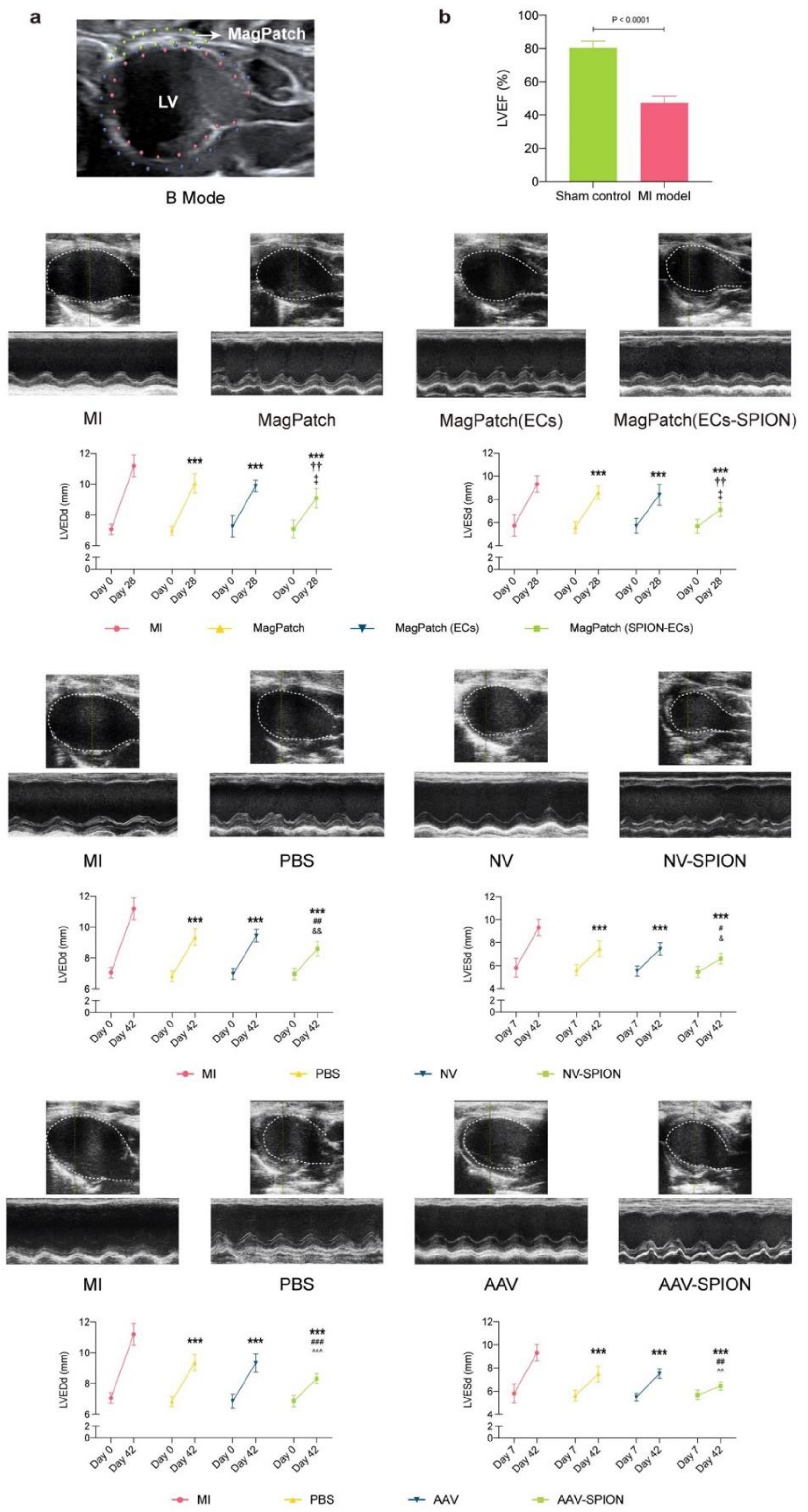

Figure S17. Echocardiographic assessment after epicardial implantation of MagPatch (ECs-SPION) and intravenous injection of NVs, NVs-SPION, Ad and Ad-SPION. a, Representative echocardiographic B-mode image presenting the MagPatch implanted onto the epicardium of an infarcted heart. **b**, Baseline LVEF was determined after 3 days post-surgery. The green dots indicated the edge of implanted MagPatch. The pink dots indicated the edge of

endocardium. The blue dots indicated the edge of epicardium. **c**, Representative echocardiographic M-mode images at day 28 for MI, MagPatch, MagPatch (ECs) and MagPatch (ECs-SPION) groups. Quantitative analysis of LVEDd and LVESd (n = 5). **d**, Representative echocardiographic M-mode images at day 42 and quantitative analysis of LVEDd and LVESd (n = 5). **e**, Representative echocardiographic M-mode images at day 42 and quantitative analysis (bottom) of LVEDd and LVESd (n = 5). The data were expressed as mean ± standard deviation and analyzed using One-way ANOVA followed by Bonferroni post hoc test. $^{***}p<0.001$ compared with MI group; $^{\dagger\dagger\dagger}p<0.001$ and $^{\dagger}p<0.05$ compared with MagPatch group; $^{\ddagger\ddagger\ddagger}p<0.001$ and $^{\ddagger}p<0.05$ compared with MagPatch (ECs) group; $^{\#\#\#}p<0.001$, $^{\#\#}p<0.01$ and $^{\#}p<0.05$ compared with PBS group; $^{\&\&}p<0.01$ and $^{\&}p<0.05$ compared with NVs group; $^{\wedge\wedge\wedge}p<0.001$ and $^{\wedge\wedge}p<0.01$ compared with NVs group. MI, myocardial infarction; SPION, Superparamagnetic iron nanoparticle; ECs, endothelial cells; LVEF, left ventricular ejection fraction; LVFS, left ventricular fraction shortening; NVs, nanovesicles; Ad, adenovirus.

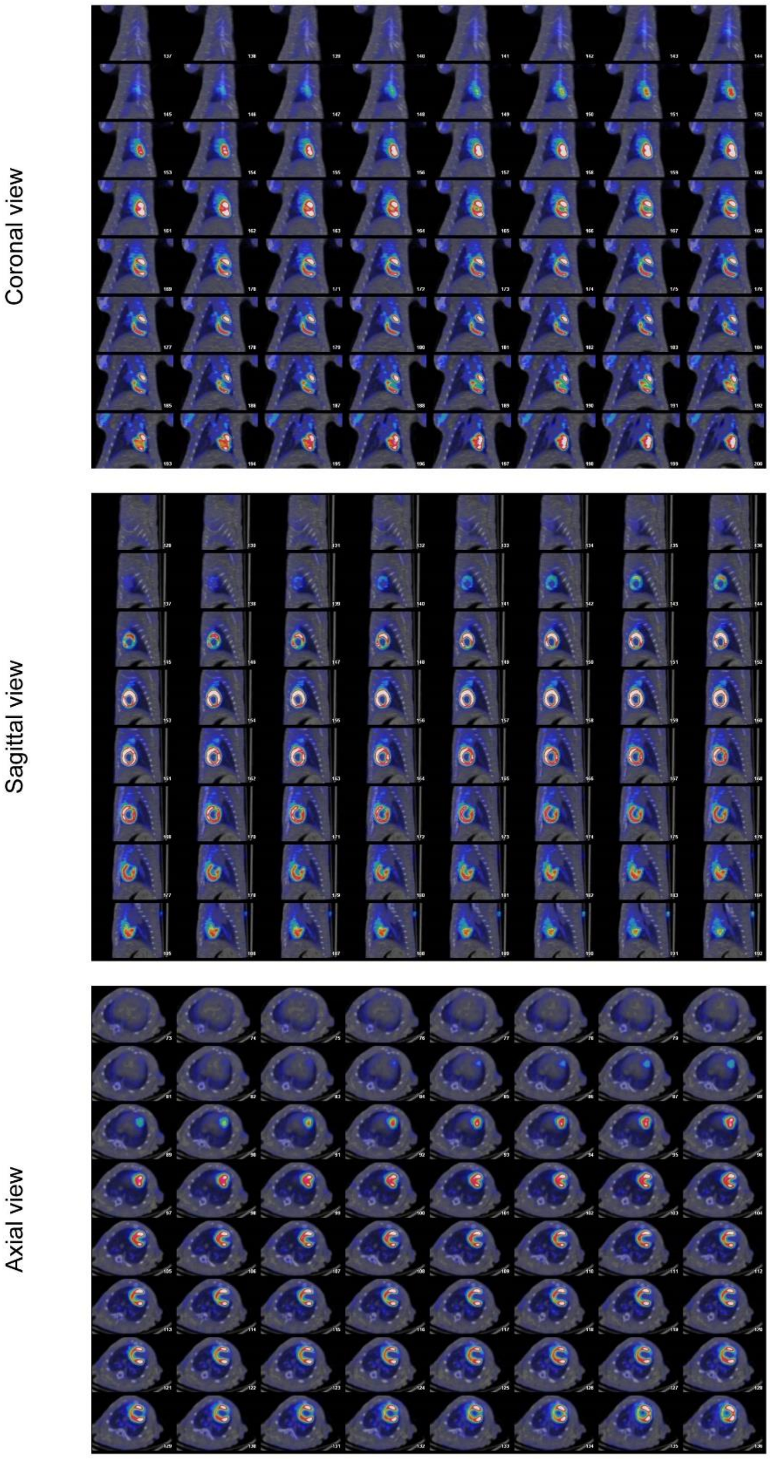

Figure S18. Assessment of the infarcted size and SUV in three axes for MI group. Representative ^{18}F-FDG PET-CT image from MI group. MI, myocardial infarction.

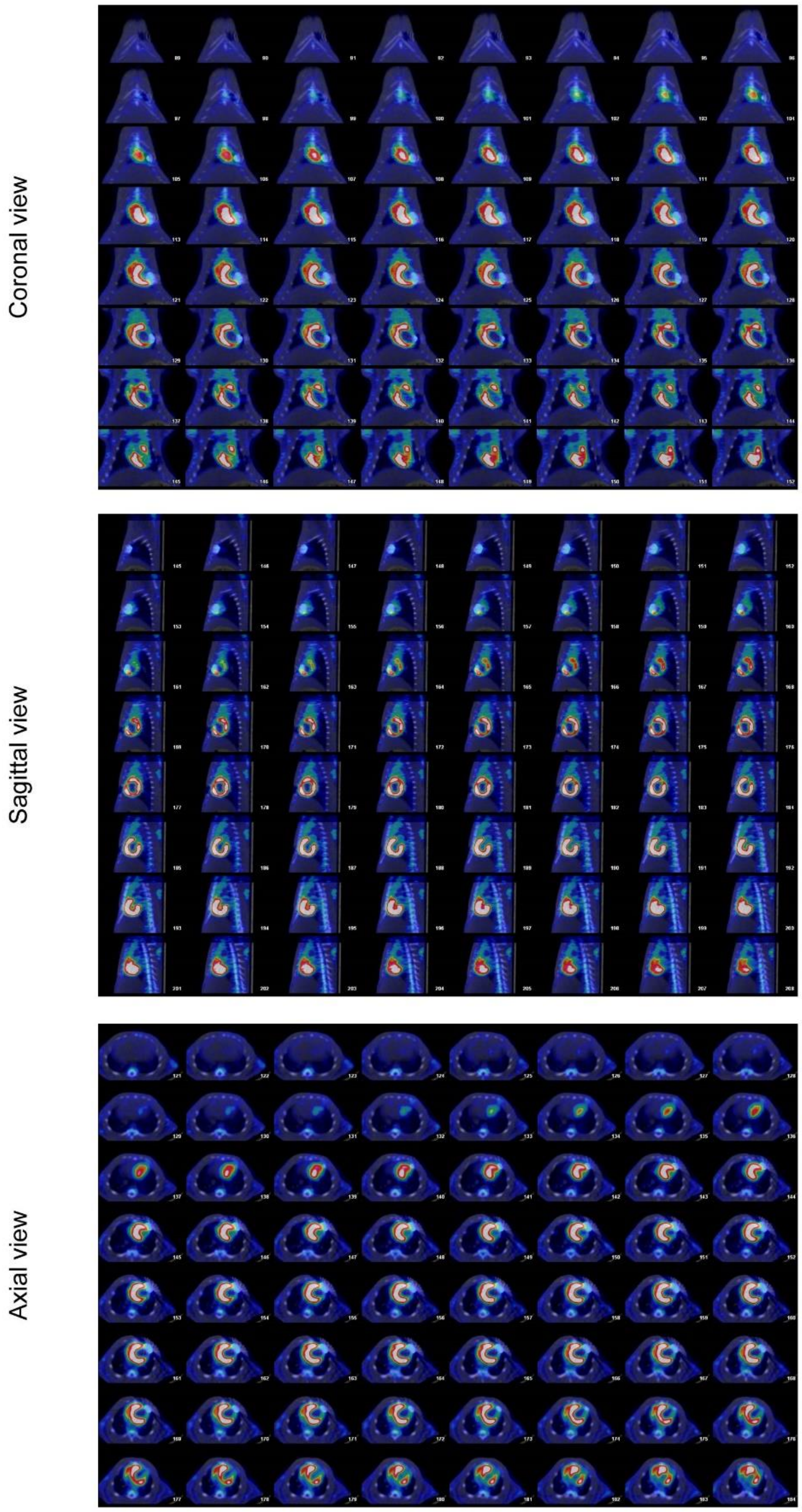

Figure S19. Assessment of the infarcted size and SUV in three axes for MagPatch group. Representative ^{18}F-FDG PET-CT image from MagPatch group.

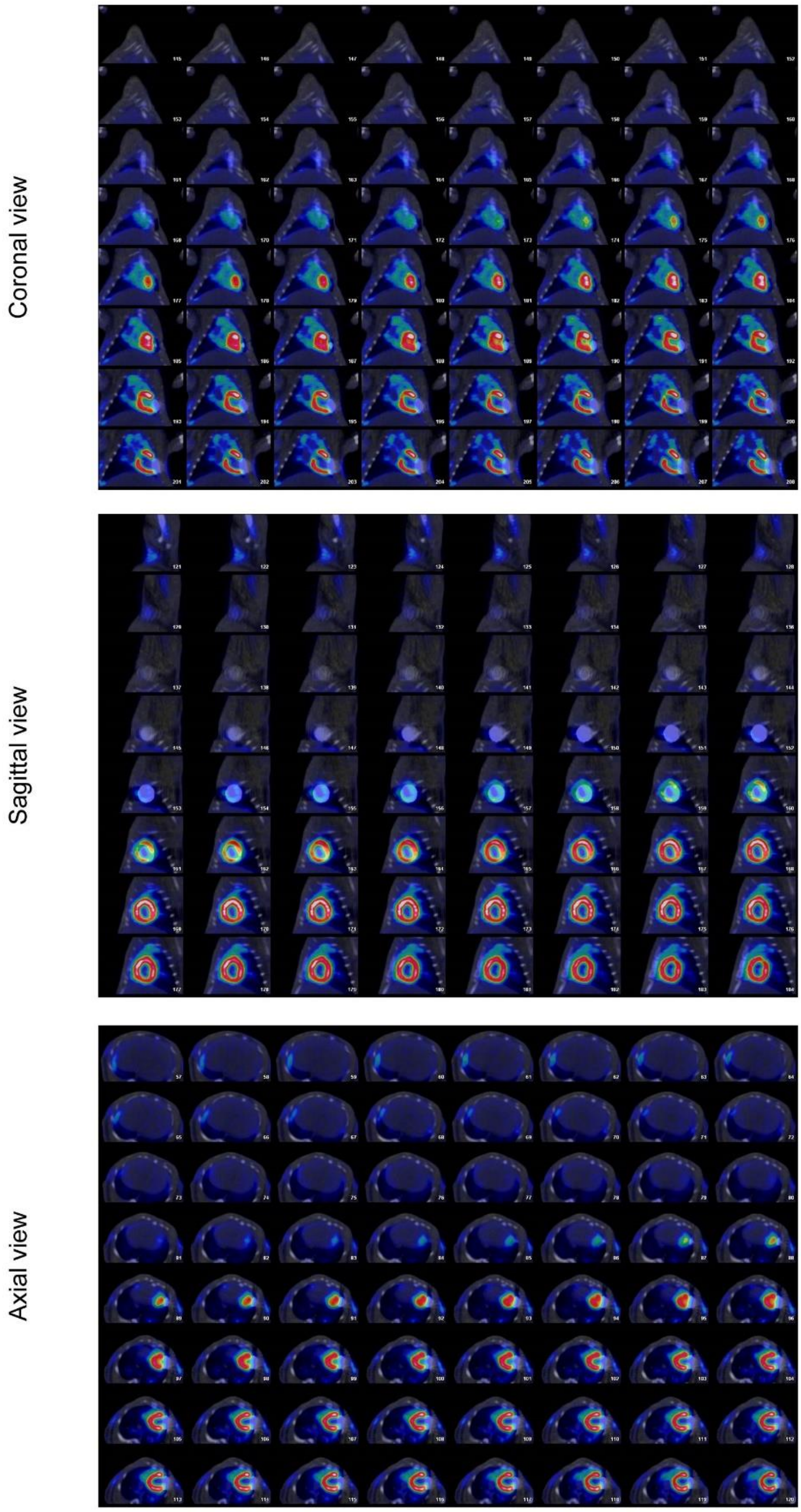

Figure S20. Assessment of the infarcted size and SUV in three axes for MagPatch (ECs) group. Representative ^{18}F-FDG PET-CT image from MagPatch (ECs) group. ECs, endothelial cells.

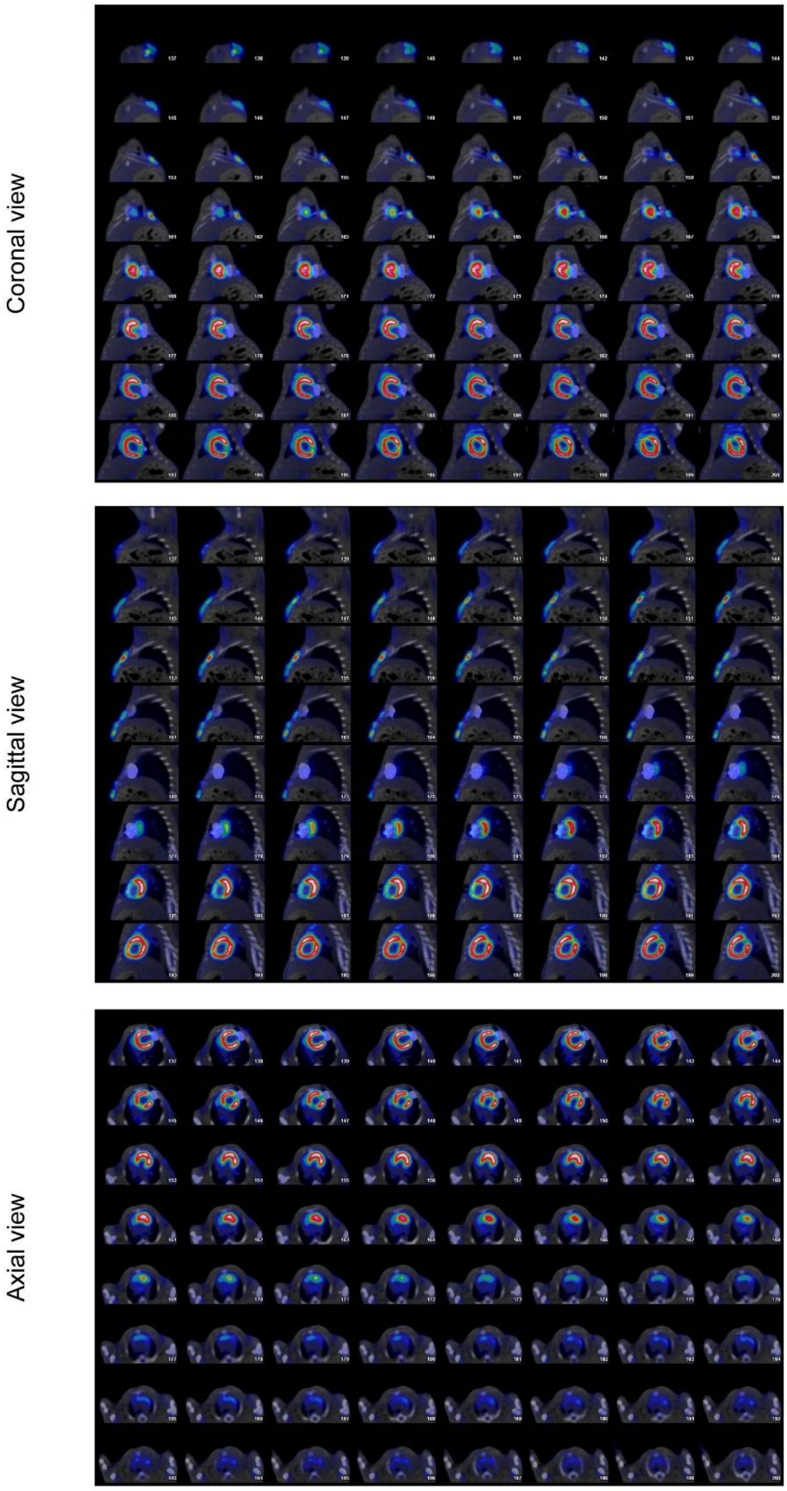

Figure S21. Assessment of the infarcted size and SUV in three axes for MagPatch (ECs-SPION) group. Representative ^{18}F-FDG PET-CT image from MagPatch (ECs-SPION) group. SPION, Superparamagnetic iron nanoparticle; ECs, endothelial cells.

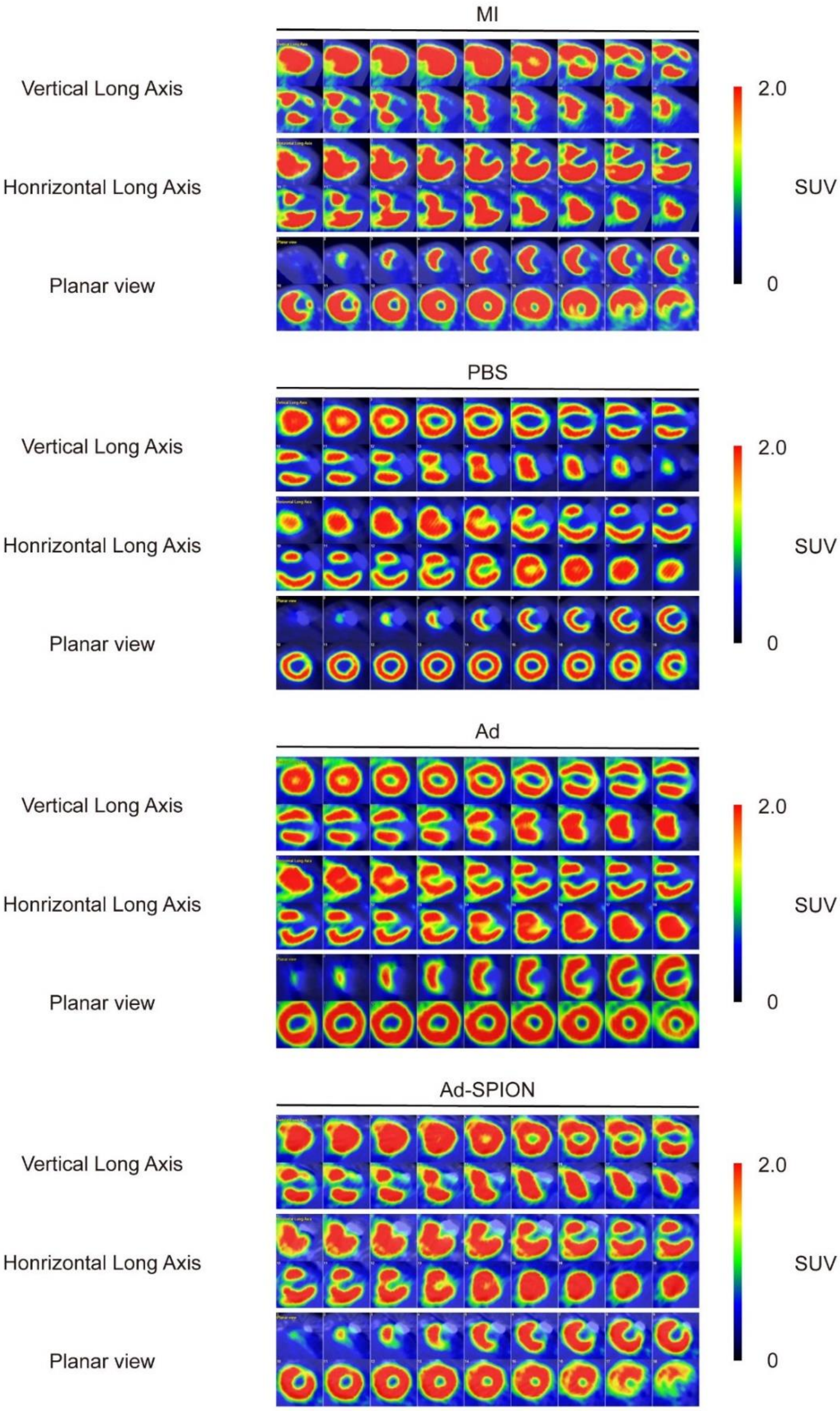

Figure S22. Assessment of the viability of LV cardiomyocytes in three axes via ^{18}F-FDG PET imaging for MI, PBS, Ad and Ad-SPION group. Eighteen contiguous images were acquired for each axis. ECs, endothelial cells; Ad, adenovirus.Supplementary Movies

Movie S1. Gross observation of MagPatch.

Movie S2. Flexibility of MagPatch.

Movie S3. Live/Dead staining of MagPatch (ECs-SPION).

Movie S4. Primary cardiomyocytes co-cultured with MagPatch.

Movie S5. MagPatch accumulated ECs-SPION efficiently in vitro.

Movie S6. Magnetic attractive force between MagPatch and ECs-SPION measured via micropipette method.

Movie S7. Permanent ligation of the LAD to establish a rat MI model and implantation of the MagPatch onto the epicardial surface.

Movie S8. Sutureless implantation of the MagPatch with assistance of hydrogel containing Fe3O4 nanoparticlesSupplementary Tables

Table S1. List of primary antibodies used in current study.

**Primary antibody**	**Company**	**Cat. No.**	**Application**	**Dilution**
Anti-mCherry	Abcam	ab125096	IF	1:500
Anti-EGFP	Abcam	ab184601	IF	1:500
Anti-CD31	Abcam	ab64543	IF	1:100
Anti-α-SMA	Abcam	ab5694	IF	1:100
Anti-α-actinin	Sigma	A7811	IF	1:500
Anti-Collagen I	Abcam	ab270993	IF	1:100
Anti-Collagen III	Abcam	ab6310	IF	1:100
Anti-isolectin B4	Vector	B-1205	IF	1:100
Anti-NG2	Abcam	ab275024	IF	1:50
Phalloidin-FITC	Sigma	P5282	IF	1:500
Anti-CD63	Invitrogen	PA5-92370	WB	1:1000
Anti-Calnexin	Invitrogen	MA5-31501	WB	1:1000
Anti-Alix	Invitrogen	MA1-83977	WB	1:1000
Anti-β-actin	Invitrogen	MA1-140	WB	1:1000
Anti-CD68	Abcam	ab283654	IHC	1:100
Anti-MPO	Abcam	ab208670	IHC	1:1000

Table S2. List of secondary antibodies used in current study.

Secondary antibody	Company	Cat. No.	Application	Dilution
Alexa Fluor® 488-conjugated Streptavidin	Jackson	016-540-084	IF	1:1000
Goat Anti-Mouse IgG H&L (Alexa Fluor® 488)	Abcam	ab150113	IF	1:500
Goat Anti-Mouse IgG H&L (Alexa Fluor® 594)	Abcam	ab150120	IF	1:500
Goat Anti-Rabbit IgG H&L (Alexa Fluor® 488)	Abcam	ab150077	IF	1:500
Goat Anti-Rabbit IgG H&L (Alexa Fluor® 594)	Abcam	ab150080	IF	1:500
HRP-conjugated Goat anti-Mouse IgG (H+L)	Invitrogen	31430	WB	1:5000

Table S3. List of primary sequences used in current study.

Gene	Forward Primer	Reverse Primer
GADPH	AGTGCCAGCCTCGTCTCATA	GTAACCAGGCGTCCGATACG
VEGF	ACGAAAGCGCAAGAAATCCC	CTCCAGGGCATTAGACAGCA
Ang-2	GCACCGCTAACCAACCAAAG	AATGCATGCTGTCCCTGTGA
VEGFR 1	ATGCGCATGAGAACACCAGA	CACAAGTTCAGCAAACCGGG
VEGFR 2	ACTCAGACGACACAGATACC	ACATCTCACCCATCCCAAC
Bax	TTGCTACAGGGTTTCATCCA	GAGTACCTGAACCGGCATCT
Bcl-2	GAGTACCTGAACCGGCATCT	GAAATCAAACAGAGGTCGCA
Caspase 3	GGCCGACTTCCTGTATGCTT	ACTGGATGAACCATGACCCG

References

[1] a) D. Lei, Y. Yang, Z. H. Liu, S. Chen, B. Y. Song, A. Shen, B. Q. Yang, S. Li, Z. Z. Yuan, Q. Qi, L. J. Sun, Y. F. Guo, H. Zuo, S. X. Huang, Q. Yang, X. M. Mo, C. L. He, B. Zhu, E. M. Jeffries, F. L. Qing, X. F. Ye, Q. Zhao, Z. W. You, *Materials Horizons* **2019**, *6* (2), 394, https://doi.org/10.1039/c8mh00937f; b) Y. Yang, D. Lei, S. X. Huang, Q. Yang, B. Y. Song, Y. F. Guo, A. Shen, Z. Z. Yuan, S. Li, F. L. Qing, X. F. Ye, Z. W. You, Q. Zhao, *Advanced Healthcare Materials* **2019**, *8* (10), https://doi.org/10.1002/adhm.201900065; c) S. Chen, T. Huang, H. Zuo, S. H. Qian, Y. F. Guo, L. J. Sun, D. Lei, Q. L. Wu, B. Zhu, C. L. He, X. M. Mo, E. Jeffries, H. Yu, Z. W. You, *Advanced Functional Materials* **2018**, *28* (46), https://doi.org/10.1002/adfm.201805108.

[2] a) S. L. Sturgill, L. G. Salyer, B. J. Biesiadecki, M. T. Ziolo, *J Vis Exp* **2022**, (189), https://doi.org/10.3791/63056; b) T. Shioya, *J Physiol Sci* **2007**, *57* (6), 327, https://doi.org/10.2170/physiolsci.RP010107.
[3] Q. Long, Z. Liu, Q. Shao, H. Shi, S. Huang, C. Jiang, B. Qian, Y. Zhong, X. He, X. Xiang, Y. Yang, B. Li, X. Yan, Q. Zhao, X. Wei, H. A. Santos, X. Ye, *Adv Sci (Weinh)* **2022**, *9* (21), e2200856, https://doi.org/10.1002/advs.202200856.
[4] a) N. H. Reynolds, W. Ronan, E. P. Dowling, P. Owens, R. M. McMeeking, J. P. McGarry, *Biomaterials* **2014**, *35* (13), 4015, https://doi.org/10.1016/j.biomaterials.2014.01.056; b) N. Liu, M. Leng, T. Yue, L. Dong, Y. Liu, Y. Peng, H. Pu, S. Xie, J. Luo, *Micromachines (Basel)* **2020**, *11* (3), https://doi.org/10.3390/mi11030249.
[5] H. Shi, T. Xue, Y. Yang, C. Jiang, S. Huang, Q. Yang, D. Lei, Z. You, T. Jin, F. Wu, Q. Zhao, X. Ye, *Sci Adv* **2020**, *6* (25), eaaz3621, https://doi.org/10.1126/sciadv.aaz3621.
[6] Q. Cai, X. Mai, W. Miao, X. Zhou, Y. Zhang, X. Liu, W. Lu, J. Zhang, N. Gu, J. Sun, *IEEE Trans Biomed Eng* **2020**, *67* (8), 2276, https://doi.org/10.1109/TBME.2019.2958683.
[7] Y. Zhang, W. Li, L. Ou, W. Wang, E. Delyagina, C. Lux, H. Sorg, K. Riehemann, G. Steinhoff, N. Ma, *PLoS One* **2012**, *7* (7), e39490, https://doi.org/10.1371/journal.pone.0039490.
[8] S. Huang, D. Lei, Q. Yang, Y. Yang, C. Jiang, H. Shi, B. Qian, Q. Long, W. Chen, Y. Chen, L. Zhu, W. Yang, L. Wang, W. Hai, Q. Zhao, Z. You, X. Ye, *Nat Med* **2021**, *27* (3), 480, https://doi.org/10.1038/s41591-021-01279-9.
[9] B. Qian, Q. Yang, M. Wang, S. Huang, C. Jiang, H. Shi, Q. Long, M. Zhou, Q. Zhao, X. Ye, *Bioact Mater* **2022**, *7*, 401, https://doi.org/10.1016/j.bioactmat.2021.05.042.
